# Metabolic profiling reveals distinct metabolic alterations in different subtypes of pituitary adenomas and confers therapeutic targets

**DOI:** 10.1186/s12967-019-2042-9

**Published:** 2019-08-28

**Authors:** Jie Feng, Hua Gao, Qi Zhang, Yang Zhou, Chuzhong Li, Sida Zhao, Lichuan Hong, Jinjin Yang, Shuyu Hao, Wan Hong, Zhengping Zhuang, Guowang Xu, Yazhuo Zhang

**Affiliations:** 1 Beijing Neurosurgical Institute, Capital Medical University, Beijing, 100050 China; 2 Neuro-Oncology Branch, National Cancer Institute, National Institutes of Health, Bethesda, MD 20852 USA; 3 CAS Key Laboratory of Separation Science for Analytical Chemistry, Dalian Institute of Chemical Physics, Chinese Academy of Sciences, Dalian, 116023 China; 4 Beijing Tiantan Hospital, Capital Medical University, Beijing, 100050 China; 5 Beijing Institute for Brain Disorders Brain Tumor Center, Capital Medical University, Beijing, 100050 China; 6 China National Clinical Research Center for Neurological Diseases, Beijing, 100050 China; 7 Department of Hepatobiliary and Pancreatic Surgery, The First Affiliated Hospital, Zhejiang University School of Medicine, Hangzhou, 310009 China; 8 University of Chinese Academy of Sciences, Beijing, 100049 China

**Keywords:** Proteomics, Metabolomics, Microarray, Pituitary adenomas, Metabolites

## Abstract

**Background:**

Pituitary adenomas are common brain tumors. Although transsphenoidal surgery are able to achieve extensive tumor removal, the rate of recurrence ranges from 5 to 20% depending on the different subtype. Further understanding of these tumors is needed to develop novel strategies to improve the prognosis of patients. But their metabolic characteristics are largely unknown.

**Methods:**

We used metabolomic, transcriptomic, and proteomic approaches to systematically investigate eight subtypes of pituitary adenomas and normal pituitary glands. By blocking IDH2, we investigate IDH2 play an inhibitory role in GH tumor cell growth and tumor secretion.

**Results:**

We found that all of the pituitary adenomas displayed downregulated glucose metabolism and glycolysis compared to normal tissues. Together with the differences in amino acids and fatty acids, we categorized these tumors into three clusters. We then re-established the reprogrammed metabolic flux in pituitary adenomas based on multiomic analyses. Take growth hormone-secreting pituitary adenomas as an example, we revealed that IDH2 is a key player in the reprogrammed metabolism of such tumors. By blocking IDH2, we confirmed that IDH2 is a potential target for the inhibition of tumor cell growth and tumor secretion.

**Conclusions:**

Our study first uncovered the metabolic landscape of pituitary adenomas and demonstrated a possible way to inhibit tumor growth by regulating aberrant metabolism.

## Background

Pituitary adenomas are common intracranial tumors with an incidence of approximately 0.1% in adults who may have clinical symptoms [1]. The actual prevalence of pituitary adenomas can be 10% or even higher as revealed by autopsy and magnetic resonance imaging in healthy people [2–4]. According to patients’ symptoms and the cellular-pathological features of tumors, pituitary adenomas are categorized into several subtypes including prolactin (PRL)-secreting pituitary adenomas (PRL-PA), growth hormone (GH)-secreting pituitary adenomas (GH-PA), adrenocorticotropic hormone (ACTH)-secreting pituitary adenomas (ACTH-PA), gonadotropin-secreting pituitary adenomas (GT-PA), and null cell pituitary adenomas (NC-PA), etc. [5]. Although transsphenoidal surgery and medications are able to achieve extensive tumor removal and a high rate of disease control, the rate of recurrence ranges from 5 to 20% depending on the different subtype [1]. Further understanding of these tumors is needed to develop novel strategies to improve the prognosis of patients.

Metabolism changes have recently been suggested as a hallmark of tumors [6] and are closely associated with tumorigenesis [7]. Unlike the biochemical and genetic characteristics [8], the metabolic alterations of pituitary adenomas are poorly understood. Pituitary adenoma cells are suggested to undergo significant metabolic reprogramming due to their aberrant hormone production, and these metabolic alterations, in turn, may influence the progression of the tumor [9]. For instance, we previously demonstrated that pituitary oncocytoma, a specific subtype of pituitary adenoma with significant mitochondrial hyperplasia, reprogrammed its metabolism by suppressing glycolysis to facilitate mitochondrial biogenesis [10]. However, the metabolic characteristics of other subtypes of pituitary adenomas are largely unknown.

In the study, we used multiple methods including metabolomics, microarray, and proteomics to investigate the metabolic alterations in eight subtypes of pituitary adenomas. In addition, we used GH-PA as an example to show the possible use of our results for therapeutic target discovery.

## Materials and methods

### Human specimen collection

All tumor samples were obtained following transsphenoidal surgery performed at Beijing Tiantan Hospital. Fresh tumor samples were stored in liquid nitrogen. 63 tissue samples include 56 pituitary adenomas and seven normal pituitary glands. 56 pituitary adenomas were classified according to the 2017 WHO classification. In the present study, 56 cases include 10 cases of corticotroph adenomas (five silent ACTH and five functional ACTH), ten cases of gonadotroph adenomas, eight cases of somatotroph adenomas, eight cases of mammosomatotroph adenoma, five cases of lactotroph adenomas, ten cases of oncocytomas, and five cases of null cell adenomas.

This study was approved by the Ethics Committee of Beijing Tiantan Hospital (KY2013-015-02). Written informed consent was obtained from all of the enrolled subjects, and the study was performed in compliance with the 1964 Helsinki declaration and its later amendments or comparable ethical standards.

### Metabolomic analysis

For the gas chromatography–mass spectrum (GC–MS) analysis, tissue samples were mixed with 600 µl methanol/water (v/v 4:1) solution containing internal standards followed by thorough homogenization in a mixed grinding apparatus (MM400, Retsch, Germany) at 25 Hz for 2 min. Supernatants were lyophilized for subsequent oximation and silylation reactions. A QP 2010 GC–MS system (Shimadzu, Japan) with a DB-5 MS fused-silica capillary column (30 m × 0.25 mm × 0.25 µm, Agilent Technologies, Santa Clara, CA, USA) was used for the metabolic profiling. A pseudotargeted GC–MS metabolomics method was established elsewhere [11–13]. A total of 280 ion features assigned to 23 groups were defined for the data collection and quantification. The system parameter settings were previously described [13]. Metabolite identities were determined based on commercial libraries (Mainlib, NIST, Wiley, and Fiehn) and an internal metabolite library.

### RNA microarray

Total RNA was extracted and purified using the mirVana™ miRNA Isolation Kit (Cat# AM1561, Ambion, Austin, TX, US) following the manufacturer’s instructions and checked for an RIN number to inspect the RNA integration using an Agilent Bioanalyzer 2100 (Agilent Technologies, Santa Clara, CA, US). Total RNA was amplified and labeled with the Low Input Quick Amp Labeling Kit, One-Color (Cat# 5190-2305, Agilent Technologies, Santa Clara, CA, US), following the manufacturer’s instructions. Labeled cRNA was purified using an RNeasy mini kit (Cat# 74106, Qiagen, GmBH, Germany). Each slide was hybridized with 1.65 µg Cy3-labeled cRNA using the Gene Expression Hybridization Kit (Cat# 5188-5242, Agilent Technologies, Santa Clara, CA, US) in a hybridization oven (Cat# G2545A, Agilent Technologies, Santa Clara, CA, US), according to the manufacturer’s instructions. After 17 h of hybridization, the slides were washed in staining dishes (Cat# 121, Thermo Shandon, Waltham, MA, US) with the Gene Expression Wash Buffer Kit (Cat# 5188-5327, Agilent Technologies, Santa Clara, CA, US), following the manufacturer’s instructions. Slides were scanned by an Agilent Microarray Scanner (Cat# G2565CA, Agilent Technologies, Santa Clara, CA, US) with the default settings: dye channel = green, scan resolution = 5 µm, PMT 100%, 10%, 16 bit. Data were extracted with Feature Extraction software 10.7 (Agilent Technologies, Santa Clara, CA, US). The raw data were normalized using the quantile algorithm and limma packages in R.

### Protein preparation and proteomic analysis

Proteins from pituitary adenoma tissues and normal pituitary glands were extracted using a total protein extraction kit (Millipore, Billerica, MA, USA). Protein concentrations were determined using the bicinchoninic acid protein assay (Pierce, Rockford, IL, USA). Proteins from each group of tissues were equally combined into a single pool as previously described [14]. A mass of 100 μg of each pooled sample was then denatured, reduced and alkylated as described in the iTRAQ protocol (Thermo Fisher Scientific, Waltham, MA, USA) and digested overnight with 0.1 µg/µl trypsin solution at 37 °C. The digested pooled samples were subsequently labeled with 113, 115, 117, 118, 119 and 121 iTRAQ tags, respectively, according to the manufacturer’s protocol (AB Sciex, Framingham, MA, USA). The tagged peptides were dried via vacuum centrifugation and combined in one tube. Strong cation-exchange (SCX) chromatography was then performed as previously described [15, 16]. In brief, pooled samples were separated on an apoly-LC SCX column (4.6 × 250 mm, 5 μm, 100 Å) using an LC 100 instrument (Eksigent Technologies, Dublin, CA, USA), and then the labeled peptides were detected by ultraviolet radiation using SPD-20 (Shimadzu, Kyoto, Japan). A total of 48 fractions were collected, dried by speed vacuum centrifugation, and combined into 10 fractions according to the SCX chromatogram. Each fraction was injected onto a desalting column (350 µm × 0.5 mm, 3 µm C18, 120 Å) and separated on an analytical column (75 µm × 150 mm, 3 µm C18, 120 Å) using an Eksigent NanoLC instrument (Eksigent, Dublin, CA, USA). The samples were separated by capillary high-performance liquid chromatography and were subsequently analyzed using a Triple TOF 5600+ system (AB Sciex).

Protein identification and proteome annotation were performed using the ProteinPilot software package, version 4.5 (Applied Biosystems), and cross-referenced against the SwissProt database (March 2013) using the Mascot 2.2 search engine (Matrix Science, London, UK). The search parameters utilized to analyze the MS/MS data included: trypsin as the digestion enzyme with a maximum of two missed cleavages allowed; fixed modifications of carbamidomethyl (C) and iTRAQ Plex (K and N-terminus); variable modifications of oxidation (M); peptide mass tolerance of ± 20 ppm; fragment mass tolerance of ± 0.1 Da; and a peptide false discovery rate (FDR) of less than or equal to 0.01.

### Cell culture

The GH3 rat pituitary cell line was purchased from the China Institute of Cell Line Resources and cultured in phenol red-free Dulbecco’s modified Eagle’s medium (DMEM, Invitrogen, China) supplemented with 10% fetal bovine serum (Gibco, Auckland, USA) in a humidified incubator at 37 °C with 5% CO_2_. IDH2-targeted shRNA (TF702283A-D) was obtained from OriGene Clone (OriGene Technologies, MD, USA). Transfection was performed using Lipofectamine 3000 (Invitrogen, Carlsbad, CA, USA) following the manufacturer’s instructions. A total of 1 µg of plasmid was used to transfect 1 × 10^6^ GH3 cells.

### Cell viability and Transwell assays

GH3 cells were transfected with shRNA or empty control vectors (noneffective 29-mer scrambled shRNA cassette in pGFP-C-shLenti vector). Cultures were adjusted to a density of 1 × 10^5^ cells/ml, and 100 µl of cell suspension was plated into each well of a 96-well plate and cultured for 0 h, 24 h or 48 h before adding 20 µl of MTS tetrazolium solution to each well with incubation for an additional 4 h. Absorbance at 490 nm was measured using an ELISA plate reader (Thermo, USA).

Cell migration was assayed on fibronectin- and Matrigel-coated polycarbonate filters in modified Transwell chambers (Corning, USA). GH3 cells (5 × 10^4^ cells) were introduced into the upper chambers. The time of incubation in the chambers was 24 h. Cells adhering to the lower membrane surface were fixed in 4% paraformaldehyde and stained with hematoxylin (Zhongshan Company, Beijing, China). The average number of migrated cells in five randomly chosen high-power fields was determined under light fields with fluorescent microscope (ZEISS, Jena, Germany). The assays were performed in triplicate.

### Quantitative reverse-transcription polymerase chain reaction (qRT-PCR)

Total RNA was extracted from GH3 cells with shRNA or empty control vectors using TRIzol reagent (Qiagen), and qRT-PCR was performed as previously described [17] with an Applied Biosystems 7500 Fast System (Life Technologies, Carlsbad, USA). The fold-change in differential expression for each gene was calculated using the comparative CT method (2−∆∆CT method) as previously described [17]. GAPDH was used as the housekeeping gene. The primers are listed in Additional file 1: Table S1).

### Statistical analysis

All data are presented as the mean ± standard error of the mean (SEM). One-way ANOVA was used for the comparisons of multiple groups. A *p* value less than 0.05 was considered statistically significant.

## Results

### Common changes of metabolism in pituitary adenomas

We collected ACTH-PA, silent ACTH-PA, GT-PA, GH-PA, PRL-PA, GH-PRL-PA, oncocytomas, NC-PA and normal pituitary glands from patients or donors and subjected these samples to metabolomic analysis. The heatmap of the metabolite profiles showed that all subtypes of pituitary adenomas were clustered together and generally different from the normal pituitary glands (Additional file 2: Figure S1). As expected, GH-PA, GH-PRL-PA, and PRL-PA formed a subclass, showing a close relationship among these three subtypes of adenomas. Intriguingly, the metabolomes of oncocytoma, GT-PA, and NC-PA seemed very similar. Silent ACTH-PA displayed widespread downregulation of metabolites compared to the other subtypes of adenomas, which is consistent with its silent clinical and biochemical manifestations [18].

**Fig. 1:**
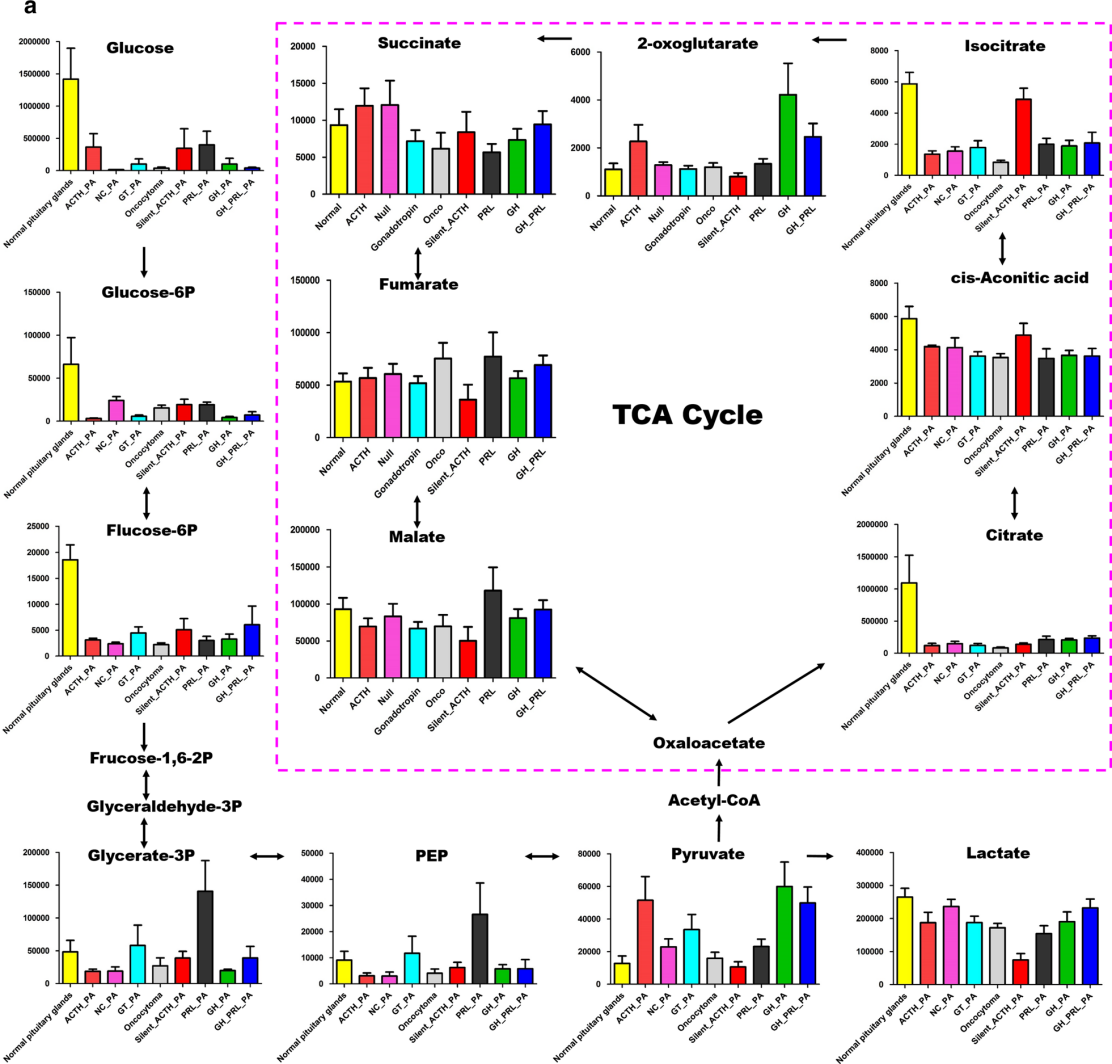

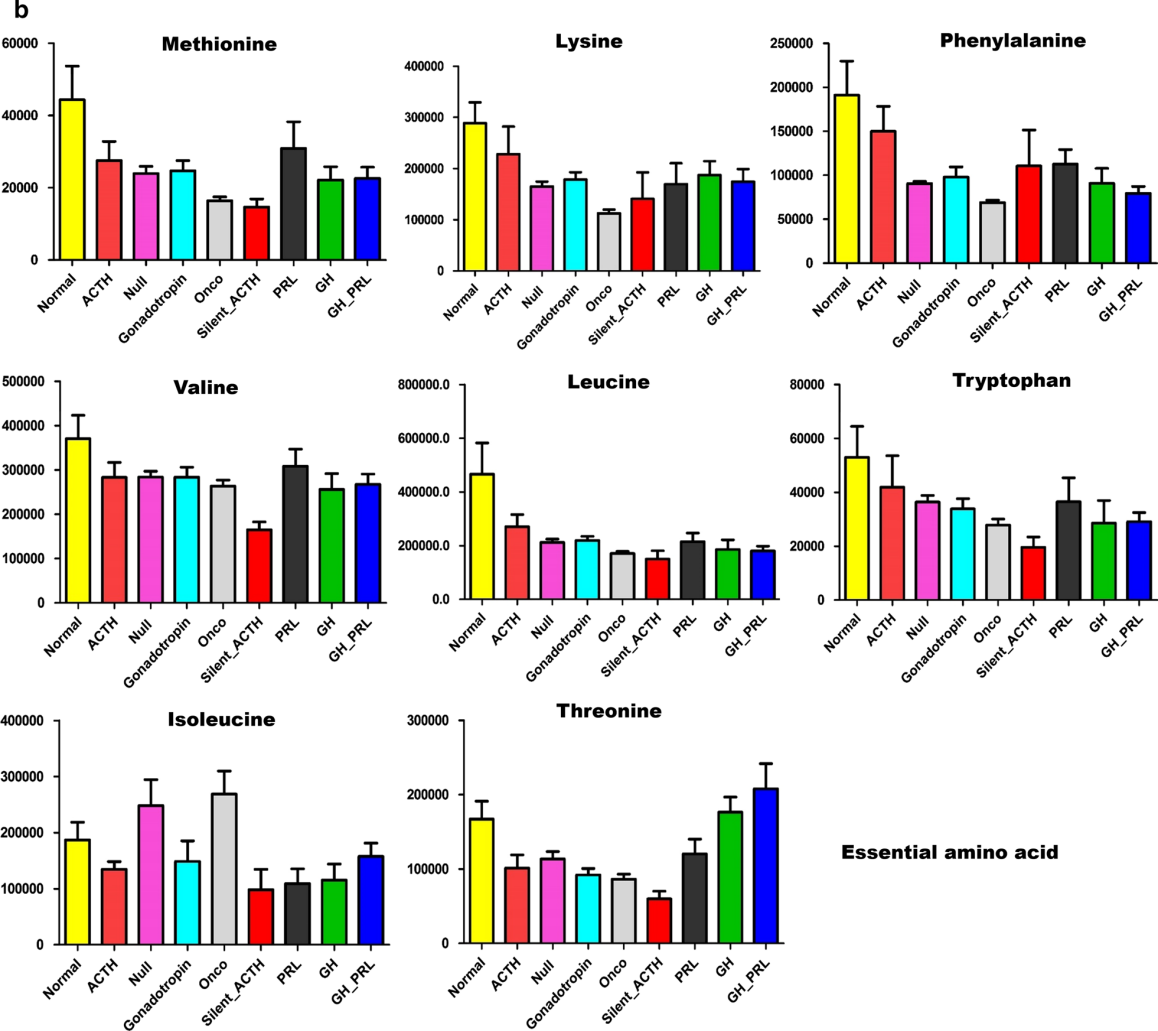

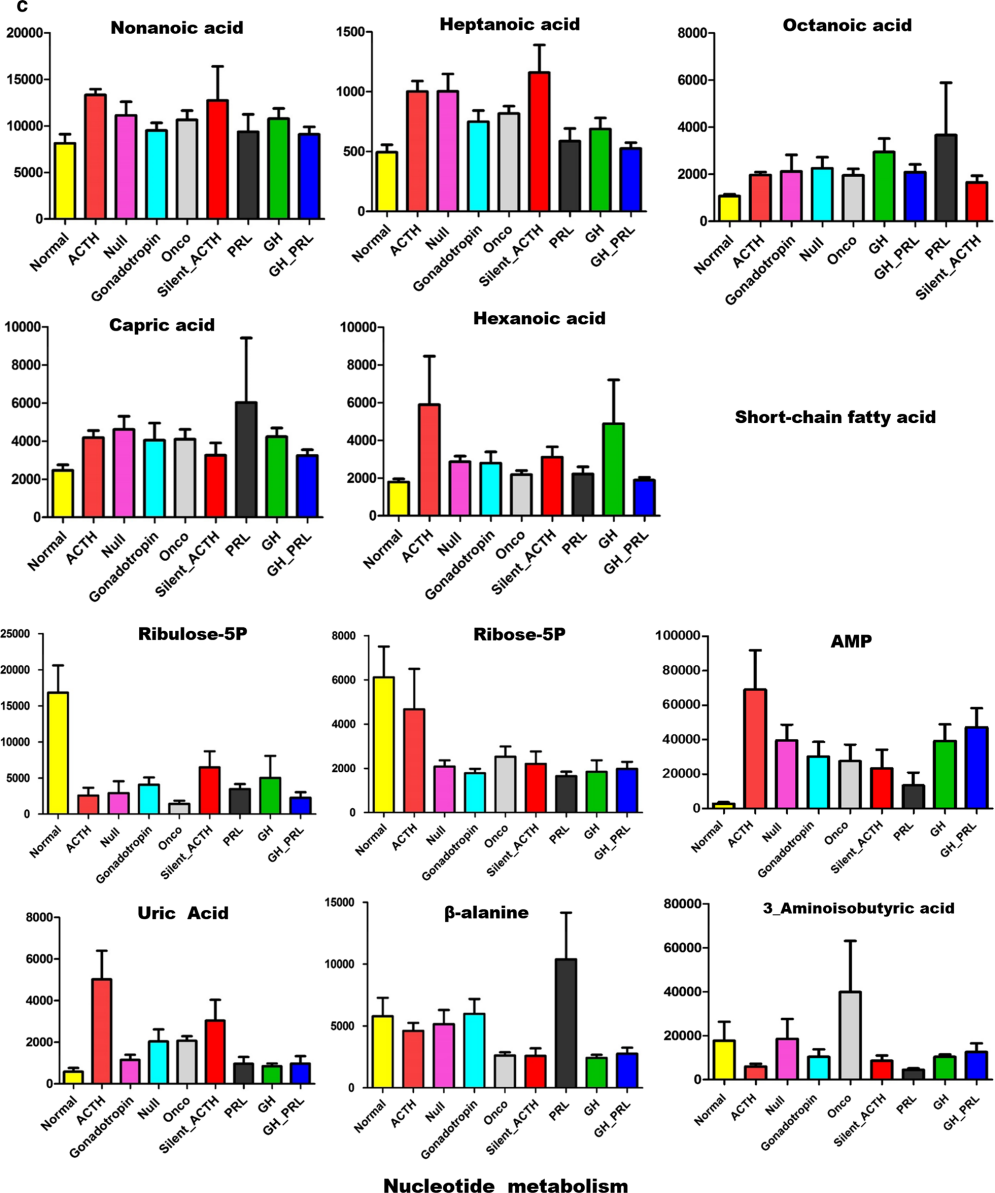
Common changes of metabolism in different subtypes of pituitary adenomas. **a** The decreased levels of tricarboxylic acid cycle and glycolysis in all subtypes of pituitary adenomas compared to normal pituitary glands; **b** the reduced essential amino acids in all subtypes of pituitary adenomas compared to normal pituitary glands; **c** the increased short-chain fatty acids and upregulated metabolites involved in nucleotide synthesis. *PRL-PA* prolactin-secreting pituitary adenomas, *GH-PA* growth hormone-secreting pituitary adenomas, *ACTH-PA* adrenocorticotropic hormone-secreting pituitary adenomas, *GT-PA* gonadotropin-secreting pituitary adenomas, *NC-PA* null cell pituitary adenomas

When further looked into the metabolites involved in different biological processes, we generally found decreased levels of tricarboxylic acid cycle (TCA) flux, glycolysis (Fig. 1a), and essential amino acids (Fig. 1b). However, the metabolites involved in nucleotide metabolism and short-chain fatty acids (Fig. 1c) were upregulated compared to that in the normal pituitary tissues.

### Energy metabolism changes in pituitary adenomas

In terms of energy metabolism, all eight subtypes of adenomas studied showed reduced levels of glucose, glucose-6-P, and fructose-6-P (Fig. 1a). Phosphoenolpyruvate (PEP) was also decreased in all samples except GT-PA and PRL-PA, while no significant reductions in pyruvate and lactate levels were observed. These findings suggested that glucose uptake is inhibited in pituitary adenomas, seemingly contradictory to Warburg’s effect. In addition, we noticed that TCA cycle-related metabolites were generally recovered downstream of isocitrate, implying that anaplerosis of glutamate might be enhanced to supplement the TCA cycle flux (Additional file 3: Figure S2). In agreement, the level of glutamate was significantly higher in GH-PA and GH-PRL-PA (p < 0.05), which also showed higher levels of alpha-ketoglutarate (α-KG) than those in normal pituitary glands (p < 0.05) (Additional file 4: Table S2). Therefore, decreased energy metabolism was a common alteration in pituitary adenomas.

### Anabolism metabolic alterations in pituitary adenomas

The levels of essential amino acids were predominantly decreased. In particular, methionine, valine, and leucine were downregulated in all subtypes of pituitary adenomas. The abundance of lysine, tryptophan, and phenylalanine was also reduced except in ACTH-PA (Fig. 1b). Furthermore, the levels of pyrophosphate, zymosterol, and adenosine-5-monophosphate were increased, while those of uric acid was upregulated or unchanged in pituitary adenomas (Fig. 1c), suggesting enhanced nucleotide metabolism. The levels of short-chain fatty acids such as nonanoic acid, heptanoic acid, octanoic acid, capric acid, and hexanoic acid were mostly increased, especially in ACTH-PA and GH-PA (Fig. 1c). Although the detailed relationship is unclear, changes in amino acids, nucleotides, and fatty acids are suggested to be connected with the endocrinal function of these cells.

### Integration of the transcriptome and metabolome in pituitary adenomas

To further understand the genetic regulation of metabolic alterations in pituitary adenomas, we performed RNA microarray in the eight subtypes of adenomas, with normal pituitary glands as the controls. The heatmap showed similar clustering of these adenomas compared to the metabolome (Additional file 5: Figure S3). Notably, the two clusters (Cluster 1: PRL-PA/GH-PA/GH-PRL-PA, and Cluster 2: GT-PA/NC-PA/oncocytoma) were again automatically formed. Intriguingly, the transcriptomes of ACTH-PA and silent ACTH-PA were quite similar, though their metabolomes were distinct, suggesting that synthesis of ACTH was associated with gene expression while secretion of ACTH was more likely to be associated with cellular metabolism.

### Identification of possible targets based on integrated multiomic data in GH-PA

**Fig. 2:**
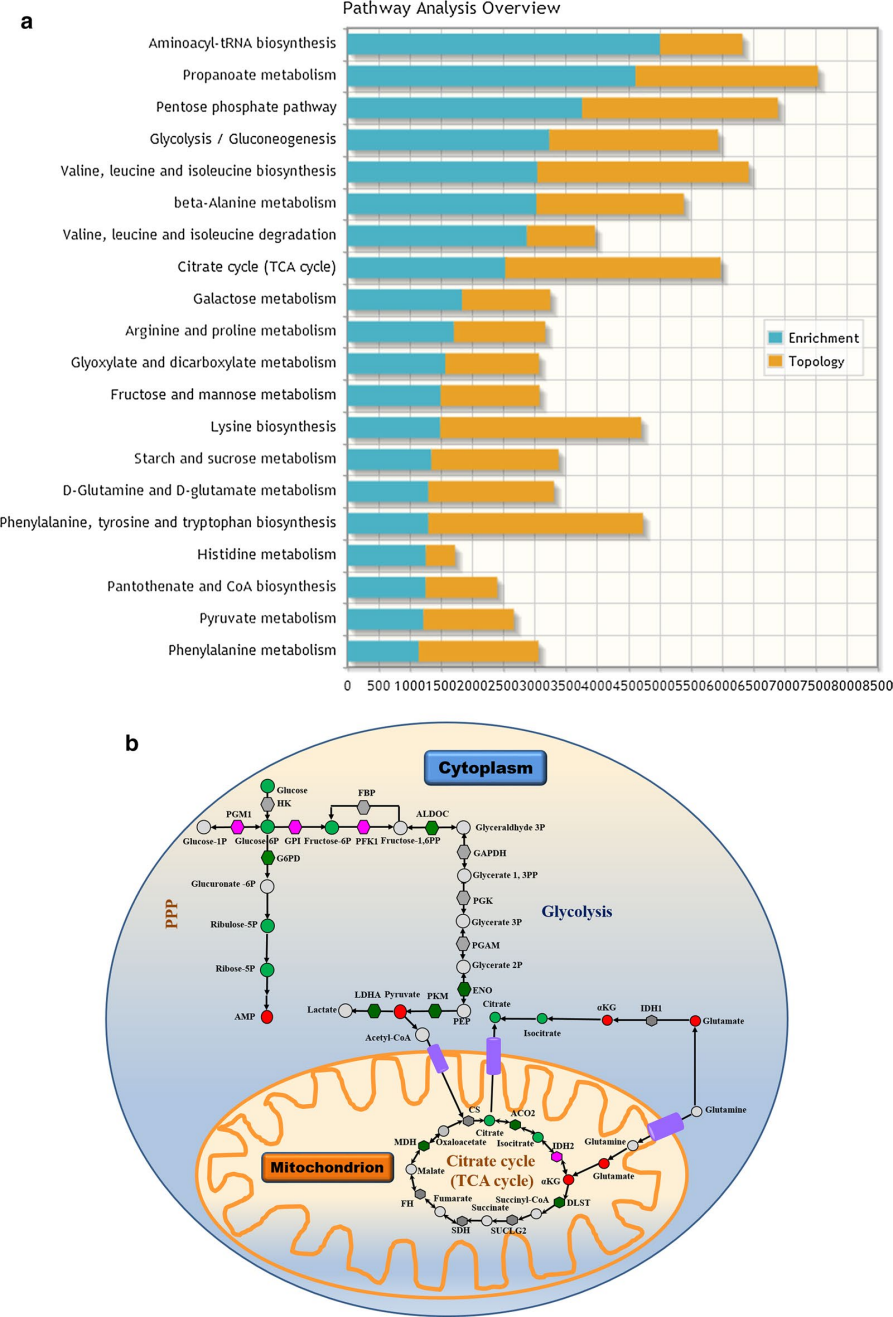
The abnormal glucose metabolism in GH-PA. **a** The stacked bars below show a summary of the joint evidence from enrichment analysis and topology analysis. The top 20 enriched pathway was showed. Blue bar (enrichment) shows joint-enrichment of both the differentially expressed proteins and metabolites in a particular pathway. Orange bar (topology) indicates a given protein or metabolite plays an important role in a biological response based on its position within a pathway. **b** The metabolites and proteins in glycolysis/gluconeogenesis pathway, pentose phosphate pathway and citrate cycle pathway was identified in GH adenoma

Since subtype-specific metabolic reprogramming may provide novel targets for intervention in the corresponding tumors, we performed a proteomic analysis in the eight subtypes of adenomas, with normal pituitary glands as the controls. The integrated results of the metabolomics and proteomics analyses in the eight subtypes of adenomas showed several changes in metabolic pathways based on the enrichment and topology analyses (Additional file 6: Table S3). Among these, glucose metabolism and arginine/proline metabolism pathways were found to be GH-PA-specific when compared to the other subtypes of pituitary adenomas (Fig. 2a and Additional file 6: Table S3). The integrated results of the metabolome and proteome analyses in GH-PA implied that three parts of the glucose metabolism pathway were significantly altered (Fig. 2b). We inferred that the overexpression of IDH2 was biologically significant, with downregulated levels of isocitrate and upregulated levels of α-KG. Therefore, we hypothesized that IDH2 inhibition might be effective for limiting the growth of GH-PA cells.

### IDH2 inhibition suppress tumor growth and migration in GH-PA

**Fig. 3:**
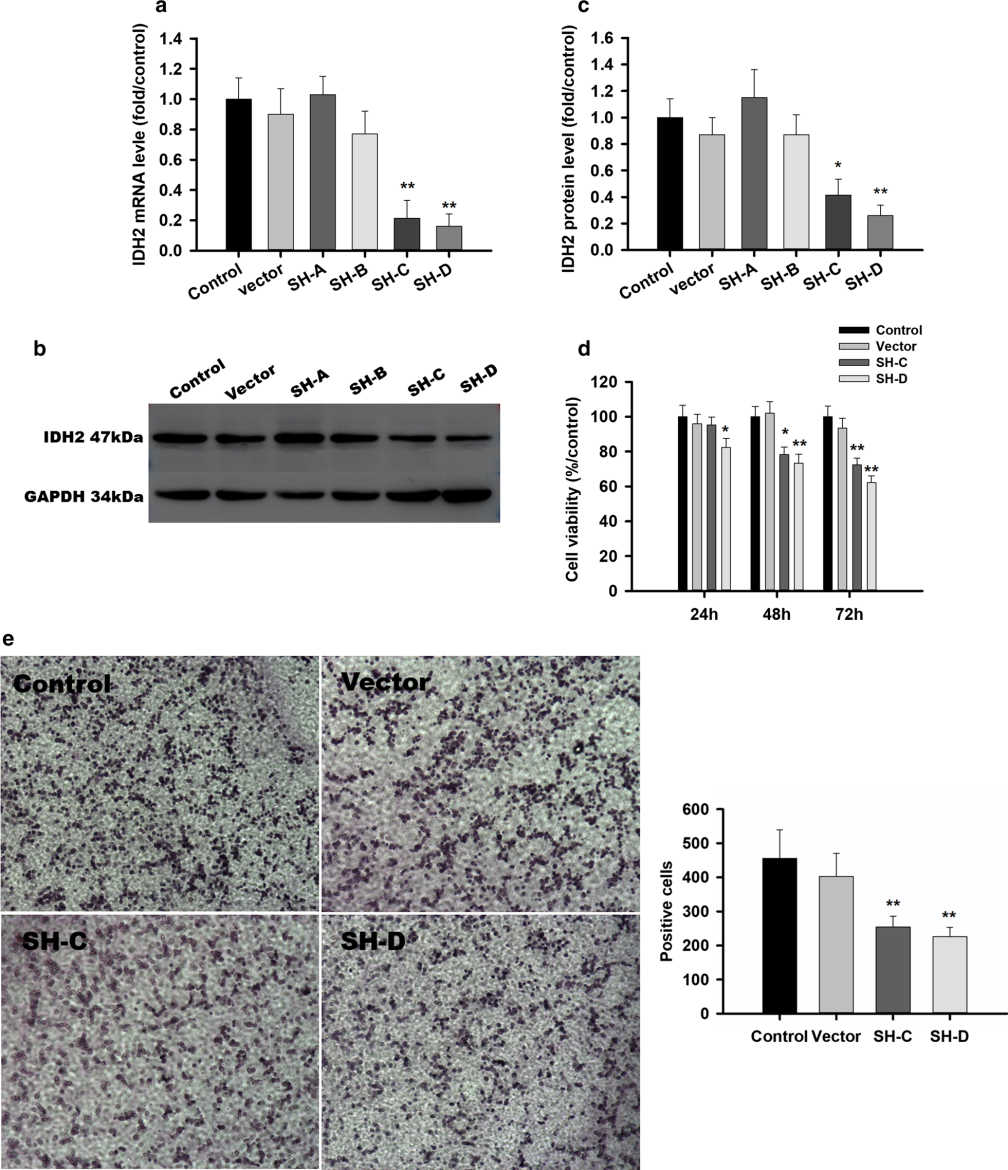
IDH2 inhibition suppress tumor growth and migration in GH-PA. **a** IDH2 mRNA levels were 103 ± 21.5%, 77.4 ± 15.2%, 21.3 ± 11.7% and 16.2 ± 8.3% of that in the control group, respectively, after RNAi in GH3 cells. **b** IDH2 protein levels were identified to be decreased after using shRNA C plasmid and shRNA D plasmid. **c** IDH2 protein levels were 115 ± 21.0%, 87.0 ± 15.0%, 41.3 ± 12.0% and 26.0 ± 8.0% of that in the control group, respectively, after RNAi in GH3 Cells. **d** The cell viability was reduced to 91.5 ± 7.1%, 77.2 ± 6.8% and 71.8 ± 7.3% after 24 h, 48 h and 72 h of shRNA C plasmid blockage and 83.9 ± 7.6%, 72.4 ± 8.1% and 64.6 ± 5.9% after 24 h, 48 h and 72 h of shRNA D plasmid blockage in GH3 cells. **e** The average number of migrated cells was reduced to 254 ± 32 and 226 ± 27 from 456 ± 84 after shRNA C and D plasmid blockage in GH3 cell lines. All assays were performed in triplicate. **Compared to control, p < 0.01. *Compared to control, p < 0.05

To test the therapeutic effects of IDH2 inhibition in GH-PA, we used four IDH2 shRNA plasmids (SH-A/B/C/D) to block IDH2 expression in GH3 cells. The IDH2 mRNA levels (SH-A/B/C/D) were 103 ± 21.5%, 77.4 ± 15.2%, 21.3 ± 11.7% and 16.2 ± 8.3%, respectively, of that in the control group 72 h after transfection, as shown in Fig. 3a. The IDH2 protein levels as determined by Western blotting showed a similar trend (Fig. 3b, c). Therefore, SH-C and SH-D were selected for the further experiments. As expected, cell viability was inhibited 3 days after IDH2 knockdown (Fig. 3d). The average number of migrated cells was reduced after IDH2 SH-C and SH-D blockage in GH3 cell lines in Fig. 3e.

### IDH2 inhibition suppress exocrine function and the expression of several key genes in GH-PA

**Fig. 4:**
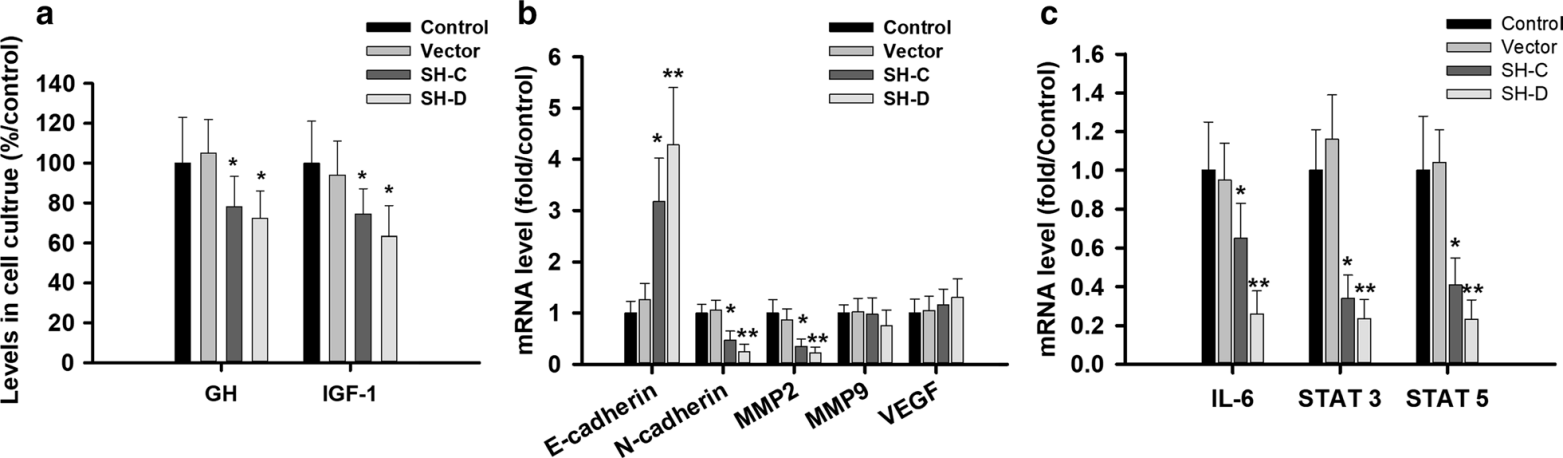
IDH2 inhibition decreased exocrine function and the expression of several key genes. **a** The levels of growth hormone were 78 ± 15.3 and 72.3 ± 13.7 mIU/m and the levels of IGF-l 74.5 ± 12.7 and 63.4 ± 15.3 mIU/m after shRNA C and D plasmid blockage in GH3 cell lines. **a** The mRNA of E-cadherin in shRNA C and D plasmid knockdown group was 3.18 and 4.28 folds of that in control group, and N-cadherin 0.47 and 0.24 folds, and MMP2 0.35 and 0.22 folds. **c** The mRNA of IL-6 in shRNA C and D plasmid knockdown group was 0.65 and 0.26 folds of that in control group, and STAT3 0.34 and 0.24 folds, and STAT5 0.41 and 0.23 folds. All assays were performed in triplicate. **Compared to control, p < 0.01. *Compared to control, p < 0.05

The exocrine function of GH3 cells was downregulated, and decreased levels of GH and insulin-like growth factor 1 (IGF-1) (Fig. 4a) were observed 3 days after IDH2 knockdown. We further detected the mRNA levels of some key GH-related and migrated-related genes, and revealed that all these genes were dramatically inhibited, including MMP2, N-cadherin, IL-6, STAT3 and STAT5 (Fig. 4b, c). These findings suggest that targeting IDH2 could reduce both the viability and functional activity of GH-PA cells.

## Discussion

Pituitary adenomas have traditionally been classified according to their produced and secreted hormones. Both hormone production and tumorigenesis are associated with metabolic reprogramming, but their relationship is poorly understood. In the present study, we comprehensively investigated the metabolism of eight common subtypes of pituitary adenomas using multiomic approaches including metabolomic, transcriptomic, and proteomic analyses.

Contradictory to the commonly held belief that most tumors display the Warburg effect [19], we revealed that pituitary adenomas actually show reduced glucose metabolism, including both glycolysis and glucose oxidation. This finding may explain the fact that the majority of pituitary adenomas are benign tumors with relatively low proliferation rates. Although some pituitary adenomas showed higher glucose uptake, as evidenced by ^18^F-fluorodeoxyglucose (FDG) positron emission tomography/computed tomography (PET/CT), the absolute incidence of pituitary FDG uptake is rare (less than 0.1%, as shown by Jeong et al. [20]). This rate is far less than the prevalence of pituitary adenomas in the general population [2], implicating that a considerable portion of pituitary adenomas do not show upregulated glucose uptake. However, positive PET scans are related to the tumor size and many patients with pituitary adenomas show increased uptake of FDG [21]. The discrepancy between PET/CT findings and our metabolomic study may be due to the distinct methodologies, which might reflect different aspects of glucose metabolism. Compared to PET/CT, our ex vivo approach demonstrated cellular glucose metabolism in a more natural condition. Intriguingly, we noticed downregulated levels of essential amino acids, the uptake of which is believed to be more specific to tumor cells than FDG [22]. However, our results may not support using radioactive amino acid analogs for PET scans in pituitary adenoma detection. The low levels of essential amino acids are also consistent with the benign behavior of these tumors.

We also studied the differences in metabolism among various types of pituitary adenomas. In general, PRL-PA, GH-PA, and GH-PRL-PA showed increased levels of glutamine and α-KG, suggesting powerful anaplerosis of glutamine. Alanine and pyruvate were increased, suggesting that pyruvate was repurposed to alanine synthesis from ATP production, as has previously been found in oncocytoma [10]. Citruline and ornithine were also found to be increased in these pituitary adenomas. In addition, NC-PA, GT-PA, and oncocytomas displayed similar metabolic patterns such as enhanced nucleotide metabolism. Silent ACTH-PA was unique since most of its metabolites were decreased except short-chain fatty acids, which were upregulated or unchanged. ACTH-PA displayed dramatic elevation of pyruvate, uric acid, and adenosine-5-monophosphate, though the biological significance of these changes is unknown.

Through the metabolomic analysis, together with the results of the RNA-seq, we identified three clusters of pituitary adenomas. Although silent ACTH-PA is a metabolically “cold” tumor, its transcriptome is the closest to that of ACTH-PA among all the subtypes of pituitary adenomas. It is thus reasonable to categorize the two subtypes as a third cluster. The clustering of certain subtypes of pituitary adenomas may be related to the similarity of their tumorigenesis. Both oncocytomas and NC-PA are nonfunctioning tumors and are clinically inactive. The two subtypes are suggested to originate from pluripotent precursor cells capable of producing various hormones at least at the mRNA level [23]. In addition, previous studies have shown that both oncocytomas and NC-PA could produce GTs [24, 25]. A recent study using highly sensitive immunohistochemistry also showed that all NC-PA actually express α- and β-GT subunits, suggesting that NC-PA is actually GT-PA in origin [26]. Taken together, this evidence agrees with our clustering of GT-PA/NC-PA/oncocytoma based on our metabolomic and transcriptomic findings. In terms of the adenomas in Cluster 1, GH-PA and GH-PRL-PA have already been classified as adenomas producing GH according to the latest classification of pituitary adenomas [5]. PRL-PA occasionally express GH, and most importantly, all Cluster 1 adenomas can be differentially defined by pituitary-specific transcription factor 1 (Pit-1) [5]. Therefore, our categorization based on metabolomics is consistent with and provides further support for the current understanding of pituitary adenomas.

We have to admit that our study contains the limit. It is largely due to the fact that these samples were obtained between 2015 and 2016, and were classified based on the last version of WHO guidelines, which differentiates null-cell adenomas and oncocytomas from gonadotroph adenomas by staining of gonadotropins. However, the latest version of WHO classification emphasizes the concept that lineage-specific transcription factors should be considered when make classification. In this case, some oncocytomas and null cell adenomas is actually gonadotroph adenomas with positivity of transcription factors SF-1. Unfortunately, since most pituitary adenomas are quite small in size, there are not enough samples left for further test of SF-1 expression to make accurate classification. However, our data of transcriptome and metabolome showed that gonadotroph adenoma, null cell adenoma, and oncocytoma had very similar metabolic and RNA expression patterns, thus the conclusions of the present study are not likely to be affected. In the future, we will collect more samples to validate the results.

In addition to a deeper understanding of metabolic reprogramming in these adenomas, we further aimed to find potential targets that could be used to develop novel treatment strategies. IDH2 is encoded by the *IDH2* gene on chromosome 15q26.1 and catalyzes the same reversible reaction within the mitochondria as IDH1 outside of the mitochondria. IDH2 is known to play important roles in cellular defense against oxidative damage through its forward oxidative decarboxylation reaction and in several cellular processes, including lipogenesis and glycolysis regulation by reverse reductive carboxylation reaction [27–29]. IDH2 mediates a reversible reaction of isocitrate dehydrogenation to α-KG inside the mitochondria. The direction of this reaction largely depends on the relative Km values of the forward oxidative decarboxylation and reverse reductive carboxylation reactions as well as the relative levels of isocitrate and α-KG within the mitochondria. In certain types of normal cells such as astrocytes and myeloid cells, a high amount of isocitrate is usually produced by IDH2 for anabolic purpose [26]. In tumors, however, the anabolic process has already been maximized and mitochondrial respiration is slowed. Tumor cells maximize isocitrate and citrate syntheses for the purpose of producing more necessary fatty acids, phosphoglycerides, and cholesterol, which are required for the rapidly dividing cell to generate biological membranes through two pathways: IDH1-mediated pathway in the cytoplasm and IDH2-mediated pathway within the mitochondria.

Given the importance of IDH2 in the biogenesis of tumor cells and our findings including unchanged expression of IDH1, we speculate that IDH2 plays a crucial role in metabolic reprogramming and biological processes related to GH-PA. First, we uncovered the overexpression of IDH2 (Additional file 7: Table S4) and a decreased ratio of isocitrate levels to α-KG levels in GH-PA. These results together suggest that the reprogrammed TCA cycle enhances anabolic metabolism in GH-PA. Second, we found that IDH2 inhibition was effective for limiting the cell growth and hormone secretion of GH-PA cells in vitro. Third, the expression of several invasive related and proliferated genes including E-cadherin, N-cadherin, MMP-2, IL6, STAT3 and STAT5 in GH-PA was changed after IDH2 knockdown, though the underlying mechanism is unclear. However, IDH2 also plays key roles in normal cells. Thus, systematic inhibition of IDH2 is not possible. For therapeutic purposes, pituitary adenoma-targeted delivery is needed.

## Conclusions

In conclusion, we determined the comprehensive metabolic alterations in common pituitary adenomas using multiomic approaches and confirmed the relationship of these pituitary adenomas from a metabolic view. Accordingly, we categorized pituitary adenomas into three clusters. We also identified IDH2 as a potential target for GH-PA intervention. Our results shed light on the understanding of metabolic reprogramming in pituitary adenomas and provide a successful example for tumor treatment by regulating aberrant metabolism.

## Supplementary information

**Additional file 1: Table S1.** The primers in RT-PCR experiments.

**Additional file 2: Figure S1.** Heatmap of 56 pituitary adenomas and 7 normal pituitary glands based on different metabolites profiles, which provides an intuitive visualization of the metabolic profile data table. Each colored cell on the map corresponds to the average concentration values in a group. Each row represents a metabolite, and each column represents a group. Red and blue indicate expression levels, respectively, above and below the median. The fold changes relative to the median are represented by a color scale at the right of the figure. The subtypes of pituitary adenomas are represented by colors according to the color bar on the right. PRL-PA: prolactin-secreting pituitary adenomas, GH-PA: growth hormone-secreting pituitary adenomas, ACTH-PA: adrenocorticotropic hormone-secreting pituitary adenomas, GT-PA: gonadotropin-secreting pituitary adenomas, NC-PA: null cell pituitary adenomas.

**Additional file 3: Figure S2.** The metabolism of non-essential amino acids in different subtypes of pituitary adenomas PRL-PA: prolactin-secreting pituitary adenomas, GH-PA: growth hormone-secreting pituitary adenomas, ACTH-PA: adrenocorticotropic hormone-secreting pituitary adenomas, GT-PA: gonadotropin-secreting pituitary adenomas, NC-PA: null cell pituitary adenomas.

**Additional file 4: Table S2.** The different changed metabolites in 8 subtypes of pituitary adenomas compared to the normal pituitary glands.

**Additional file 5: Figure S3.** Heatmap of 56 pituitary adenomas and 7 normal pituitary glands based on different RNA profiles, which provides an intuitive visualization of the gene expression profiling data table. Each colored cell on the map corresponds to the average expression values in a group. Each row represents a gene, and each column represents a group. Red and blue indicate expression levels, respectively, above and below the median. The fold changes relative to the median are represented by a color scale at the right of the figure. The subtypes of pituitary adenomas are represented by colors according to the color bar on the right. PRL-PA: prolactin-secreting pituitary adenomas, GH-PA: growth hormone-secreting pituitary adenomas, ACTH-PA: adrenocorticotropic hormone-secreting pituitary adenomas, GT-PA: gonadotropin-secreting pituitary adenomas, NC-PA: null cell pituitary adenomas.

**Additional file 6: Table S3.** A summary of the protein-metabolite joint pathways from enrichment analysis and topology analysis.

**Additional file 7: Table S4.** The H-Score of IDH2 in different subtypes of pituitary adenomas.

## Data Availability

All data and material in this study are available from the corresponding author upon reasonable request.

## Abbreviations

PRL: prolactin
PRL-PA: prolactin-secreting pituitary adenomas
GH: growth hormone
GH-PA: growth hormone-secreting pituitary adenomas
ACTH: adrenocorticotropic hormone
ACTH-PA: adrenocorticotropic hormone-secreting pituitary adenomas
GT: gonadotropin
GT-PA: gonadotropin-secreting pituitary
NC-PA: null cell adenomas pituitary adenomas,
GC–MS: gas chromatography–mass spectrum
FDR: false discovery rate
DMEM: Dulbecco’s modified Eagle’s medium
SEM: standard error of the mean
qRT-PCR: quantitative reverse-transcription polymerase chain reaction
TCA: tricarboxylic acid cycle
PEP: phosphoenolpyruvate
α-KG: alpha-ketoglutarate
IGF-1: insulin-like growth factor 1
MMP2: matrix metalloproteinase 2
IL-6: interleukin 6
STAT3: Signal Transducer and Activator of Transcription 3
STAT5: Signal Transducer and Activator of Transcription 5
FDG: fluorodeoxyglucose
PET/CT: positron emission tomography/computed tomography
Pit-1: transcription factor 1
