# Supplementary material for: Metabolic profiling reveals distinct metabolic alterations in different subtypes of pituitary adenomas and confers therapeutic targets
Source: J Transl Med. 2019 Aug 28;17:291. doi: 10.1186/s12967-019-2042-9 (PMC6712670; doi:10.1186/s12967-019-2042-9)
Supplement: Supplementary file 1 — Additional file 1: Table S1. The primers in RT-PCR experiments. [file 12967_2019_2042_MOESM1_ESM.docx]

Additional table S1 The primers in RT-PCR experiments

| **Gene** | **Forward** | **Rerverse** |
| --- | --- | --- |
| E-CAD | actttggtgtgggtcaggaa | cacatgctcagcgtcttctc |
| N-CAD | agaacagggtggacgtcatt | accactgtgactagcccatc |
| MMP-2 | tgcaaccacaaccaactacg | tagagctcctggatcccctt |
| MMP-9 | tgggcaagcagtactctacc | gtcttcatgcagaggggagt |
| VEGF | caccaaagccagcacatagg | tttaactcaagctgcctcgc |
| SSTR1 | tatgcttggtgggactgtgt | tgaacatgttgaccgcatcc |
| SSTR2 | catctacttcgtggtgtgcg | taccgtccacagtcatgacc |
| SSTR5 | tctacggctttctctcggac | atcccagaagacaacaccgt |
| GAPDH | agtctactggcgtcttcacc | ccacgatgccaaagttgtca |
