# Supplementary figures and images for: Metabolic profiling reveals distinct metabolic alterations in different subtypes of pituitary adenomas and confers therapeutic targets
Source: J Transl Med. 2019 Aug 28;17:291. doi: 10.1186/s12967-019-2042-9 (PMC6712670; doi:10.1186/s12967-019-2042-9)

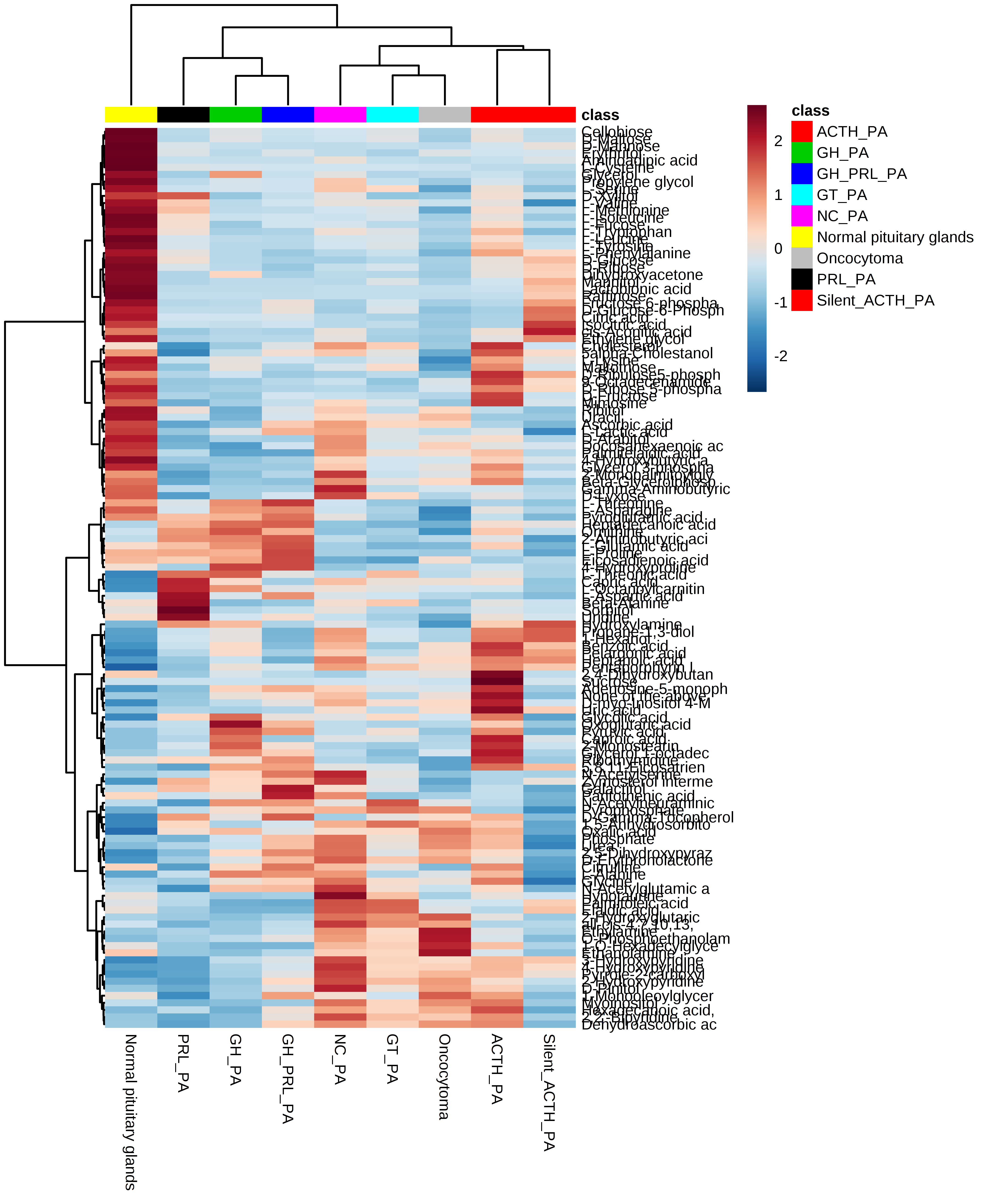

Supplement: Supplementary file 2 — Additional file 2: Figure S1. Heatmap of 56 pituitary adenomas and 7 normal pituitary glands based on different metabolites profiles, which provides an intuitive visualization of the metabolic profile data table. Each colored cell on the map corresponds to the average concentration values in a group. Each row represents a metabolite, and each column represents a group. Red and blue indicate expression levels, respectively, above and below the median. The fold changes relative to the median are represented by a color scale at the right of the figure. The subtypes of pituitary adenomas are represented by colors according to the color bar on the right. PRL-PA: prolactin-secreting pituitary adenomas, GH-PA: growth hormone-secreting pituitary adenomas, ACTH-PA: adrenocorticotropic hormone-secreting pituitary adenomas, GT-PA: gonadotropin-secreting pituitary adenomas, NC-PA: null cell pituitary adenomas. [file 12967_2019_2042_MOESM2_ESM.tiff]

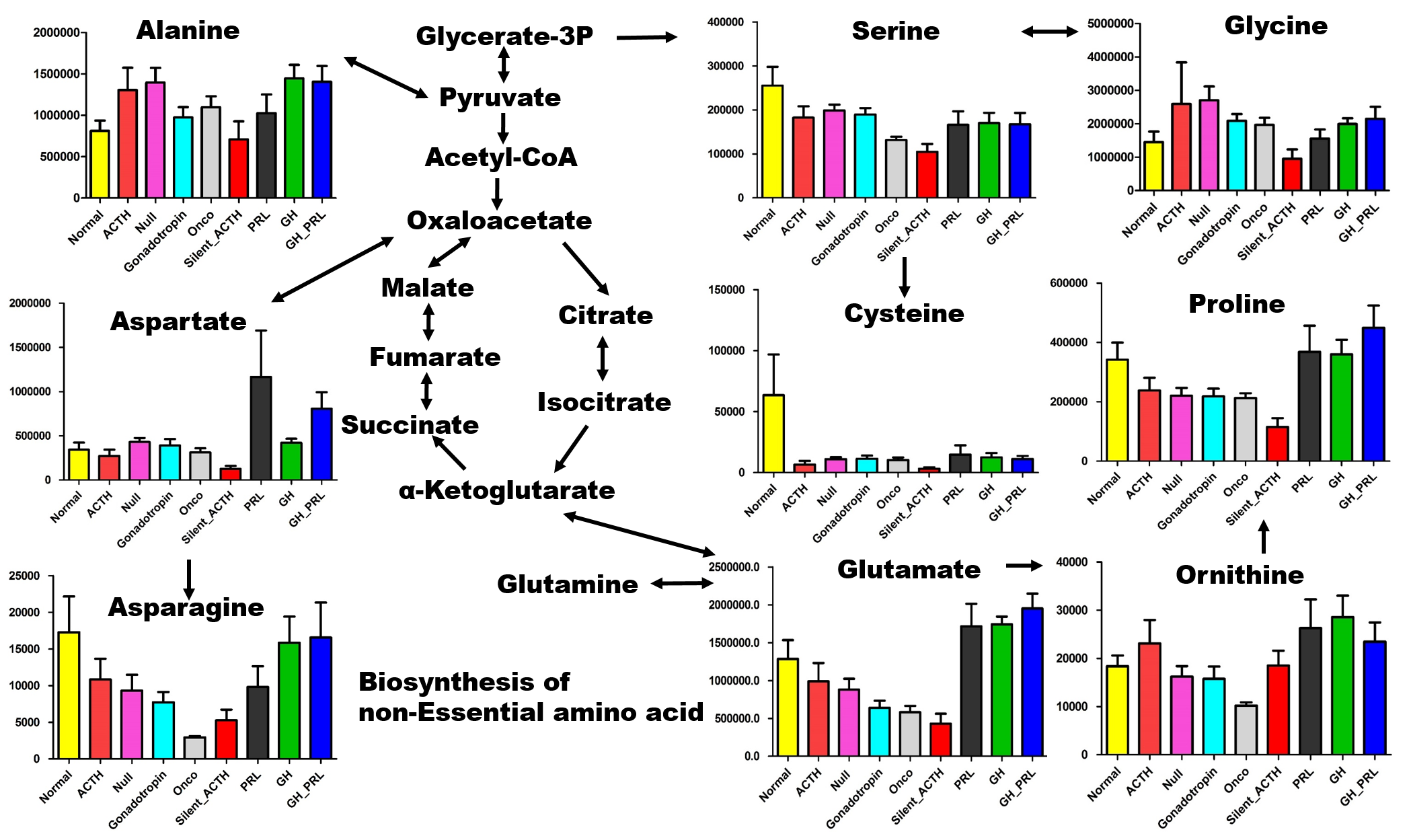

Supplement: Supplementary file 3 — Additional file 3: Figure S2. The metabolism of non-essential amino acids in different subtypes of pituitary adenomas PRL-PA: prolactin-secreting pituitary adenomas, GH-PA: growth hormone-secreting pituitary adenomas, ACTH-PA: adrenocorticotropic hormone-secreting pituitary adenomas, GT-PA: gonadotropin-secreting pituitary adenomas, NC-PA: null cell pituitary adenomas. [file 12967_2019_2042_MOESM3_ESM.tif]

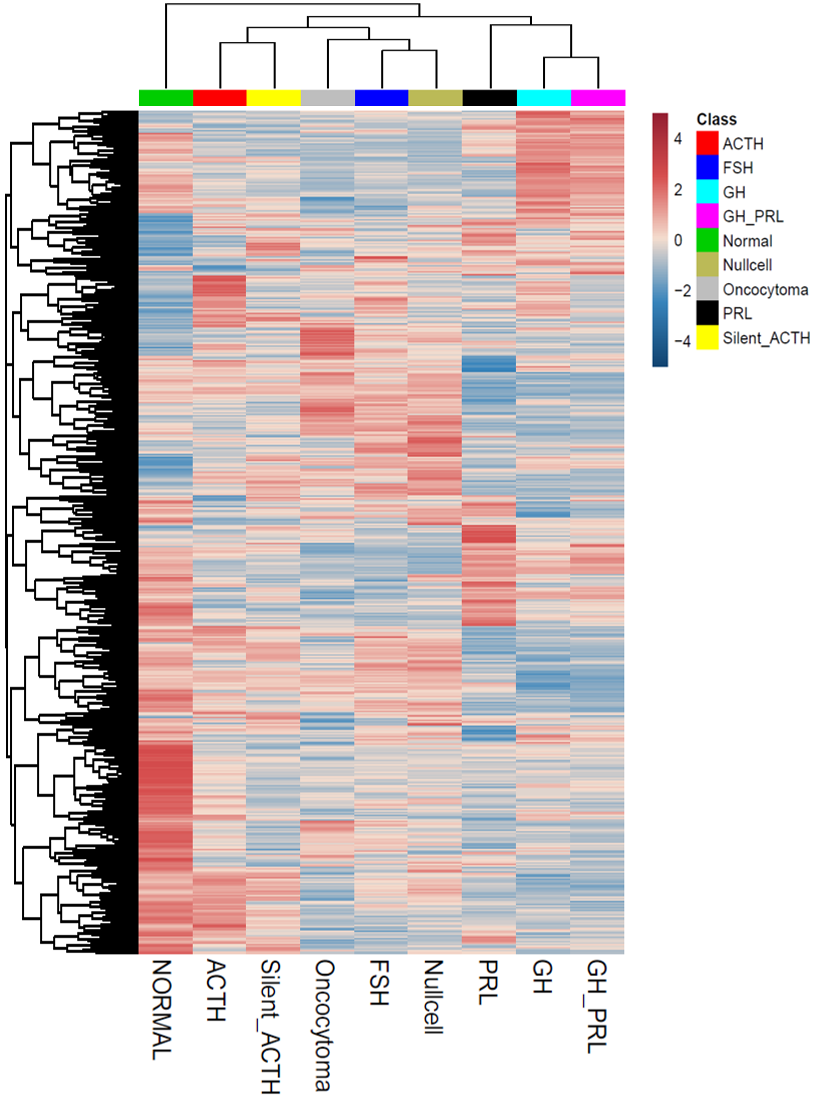

Supplement: Supplementary file 5 — Additional file 5: Figure S3. Heatmap of 56 pituitary adenomas and 7 normal pituitary glands based on different RNA profiles, which provides an intuitive visualization of the gene expression profiling data table. Each colored cell on the map corresponds to the average expression values in a group. Each row represents a gene, and each column represents a group. Red and blue indicate expression levels, respectively, above and below the median. The fold changes relative to the median are represented by a color scale at the right of the figure. The subtypes of pituitary adenomas are represented by colors according to the color bar on the right. PRL-PA: prolactin-secreting pituitary adenomas, GH-PA: growth hormone-secreting pituitary adenomas, ACTH-PA: adrenocorticotropic hormone-secreting pituitary adenomas, GT-PA: gonadotropin-secreting pituitary adenomas, NC-PA: null cell pituitary adenomas. [file 12967_2019_2042_MOESM5_ESM.tif]
