# Supplementary material for: Metabolic profiling reveals distinct metabolic alterations in different subtypes of pituitary adenomas and confers therapeutic targets
Source: J Transl Med. 2019 Aug 28;17:291. doi: 10.1186/s12967-019-2042-9 (PMC6712670; doi:10.1186/s12967-019-2042-9)
Supplement: Supplementary file 4 — Additional file 4: Table S2. The different changed metabolites in 8 subtypes of pituitary adenomas compared to the normal pituitary glands. [file 12967_2019_2042_MOESM4_ESM.docx]

| Additional Table S2 the different changed metabolites in 8 subtypes of pituitary adenomas compared to the normal pituitary glands | | | | | | | | | | | | | | | | |
| --- | --- | --- | --- | --- | --- | --- | --- | --- | --- | --- | --- | --- | --- | --- | --- | --- |
|  | Oncocytooma | | Null cell adenoma | | ACTH-Pituitary adenoma | | Gonadotrophin-Pituitary adenoma | | GH-Pituitary adenoma | | GH/PRL-Pituitary adenoma | | PRL-Pituitary adenoma | | Silent ACTH-Pituitary adenoma | |
| **name** | p  value | Ratio | p value | Ratio | p value | Ratio | p value | Ratio | p value | Ratio | p value | Ratio | p value | Ratio | p value | Ratio |
| 1,2-Propanediol | 0.032 | 0.56 | 0.487 | 0.86 | 0.253 | 0.69 | 0.495 | 0.79 | 0.728 | 1.26 | 0.125 | 0.59 | 0.317 | 0.69 | 0.570 | 0.87 |
| 1,3-Dihydroxyacetone | 0.001 | 0.121 | 0.004 | 0.170 | 0.022 | 0.227 | 0.002 | 0.200 | 0.028 | 0.266 | 0.002 | 0.157 | 0.007 | 0.169 | 0.028 | 0.25 |
| 1,3-Propanediol | 0.118 | 1.334 | 0.083 | 1.531 | 0.032 | 1.579 | 0.097 | 1.296 | 0.083 | 1.447 | 0.153 | 1.268 | 0.391 | 1.249 | 0.042 | 1.82 |
| 1,5-Anhydro-D-Sorbitol | 0.032 | 2.577 | 0.028 | 2.947 | 0.086 | 1.921 | 0.064 | 3.219 | 0.418 | 1.368 | 0.101 | 2.061 | 0.153 | 2.044 | 0.570 | 0.85 |
| 11,14-Eicosadienoic acid | 1.000 | 1.129 | 0.028 | 0.439 | 0.063 | 0.586 | 0.006 | 0.477 | 0.563 | 1.285 | 0.153 | 1.703 | 0.568 | 0.663 | 0.123 | 0.55 |
| 11-Eicosenoic acid | 0.242 | 1.331 | 0.563 | 1.081 | 0.391 | 0.744 | 0.558 | 1.081 | 0.563 | 0.833 | 0.491 | 1.162 | 0.317 | 0.575 | 0.167 | 0.75 |
| 1-Monooleoylglycerol | 0.118 | 1.294 | 0.643 | 0.895 | 0.253 | 0.587 | 0.435 | 0.691 | 0.165 | 0.739 | 0.223 | 1.020 | 0.063 | 0.495 | 0.042 | 0.64 |
| 1-O-Hexadecylglycerol | 0.005 | 3.870 | 0.015 | 1.786 | 0.317 | 1.532 | 0.380 | 1.348 | 0.002 | 0.352 | 0.001 | 0.335 | 0.046 | 0.627 | 0.372 | 0.98 |
| 2,2'-Bipyridine | 0.051 | 1.963 | 0.015 | 2.022 | 0.253 | 1.313 | 0.242 | 1.556 | 0.203 | 0.913 | 0.427 | 1.153 | 0.063 | 0.710 | 0.935 | 1.04 |
| 2,3,4-Trihydroxybutyric acid | 0.242 | 1.239 | 1.000 | 0.969 | 0.475 | 1.076 | 0.495 | 0.884 | 0.105 | 0.790 | 0.315 | 0.817 | 0.668 | 0.995 | 0.570 | 1.02 |
| 2,4-Dihydroxybutanoic acid | 0.329 | 0.564 | 0.064 | 0.482 | 0.668 | 0.893 | 0.079 | 0.468 | 0.203 | 0.473 | 0.039 | 0.379 | 0.015 | 0.392 | 0.042 | 0.35 |
| 2,5-Dihydroxypyrazine | 0.008 | 4.144 | 0.008 | 5.740 | 0.063 | 3.535 | 0.025 | 3.074 | 0.021 | 3.504 | 0.010 | 4.720 | 0.317 | 1.823 | 0.465 | 1.70 |
| 2-Aminoadipic acid | 0.001 | 0.060 | 0.037 | 0.345 | 0.007 | 0.128 | 0.001 | 0.157 | 0.001 | 0.125 | 0.001 | 0.129 | 0.003 | 0.171 | 0.004 | 0.09 |
| 2-Aminobutyric acid | 0.435 | 1.354 | 0.418 | 1.344 | 0.317 | 1.462 | 0.770 | 1.103 | 0.011 | 3.023 | 0.017 | 2.365 | 0.032 | 2.697 | 0.123 | 0.54 |
| 2-Hydroxyglutaric acid | 0.064 | 2.105 | 0.049 | 2.471 | 0.317 | 1.433 | 0.051 | 1.694 | 0.083 | 0.680 | 0.266 | 0.648 | 0.475 | 0.917 | 0.372 | 0.67 |
| 2-Hydroxyisovaleric acid | 0.770 | 0.991 | 0.563 | 0.828 | 0.568 | 0.889 | 0.696 | 1.024 | 0.418 | 0.799 | 0.368 | 0.769 | 1.000 | 1.103 | 0.062 | 0.34 |
| 2-Hydroxypyridine | 0.003 | 2.198 | 0.015 | 2.145 | 0.317 | 1.201 | 0.143 | 1.602 | 0.817 | 1.151 | 0.153 | 1.282 | 0.116 | 0.749 | 0.685 | 1.07 |
| 2-Monopalmitoylglycerol | 0.097 | 0.778 | 0.165 | 1.059 | 0.475 | 0.789 | 0.064 | 0.744 | 0.015 | 0.605 | 0.039 | 0.675 | 0.003 | 0.539 | 0.088 | 0.63 |
| 2-Monostearin | 0.329 | 1.160 | 0.643 | 1.151 | 0.015 | 1.560 | 1.000 | 0.973 | 0.165 | 1.385 | 0.081 | 1.361 | 0.391 | 1.220 | 0.223 | 1.56 |
| 3-Aminoisobutyric acid | 0.558 | 1.152 | 0.817 | 0.899 | 0.199 | 0.567 | 0.558 | 0.719 | 0.487 | 1.202 | 0.958 | 0.966 | 0.086 | 0.447 | 0.570 | 0.84 |
| 3-Hydroxybutyric acid | 0.770 | 1.849 | 1.000 | 1.746 | 0.568 | 0.874 | 0.283 | 0.478 | 0.908 | 1.159 | 0.874 | 1.162 | 0.568 | 0.837 | 0.062 | 0.39 |
| 3-Hydroxypropanoic acid | 0.558 | 1.108 | 0.908 | 0.990 | 0.391 | 1.313 | 0.696 | 0.997 | 0.418 | 1.245 | 0.427 | 0.803 | 0.568 | 0.880 | 0.291 | 0.84 |
| 3-Hydroxypyridine | 0.015 | 1.570 | 0.005 | 1.679 | 0.046 | 1.411 | 0.051 | 1.529 | 0.165 | 1.355 | 0.125 | 1.450 | 0.886 | 1.003 | 0.088 | 1.35 |
| 3-Phosphoglyceric acid | 0.380 | 0.326 | 0.247 | 0.382 | 0.475 | 0.480 | 0.770 | 0.419 | 0.487 | 0.505 | 0.791 | 0.419 | 0.116 | 3.329 | 0.808 | 1.03 |
| 4-Aminobutyric acid | 0.435 | 0.773 | 0.728 | 1.277 | 0.475 | 0.718 | 0.079 | 0.329 | 0.004 | 0.146 | 0.005 | 0.210 | 0.022 | 0.131 | 0.088 | 0.42 |
| 4-Hydroxybutyric acid | 0.001 | 0.353 | 0.015 | 0.600 | 0.046 | 0.225 | 0.001 | 0.294 | 0.001 | 0.144 | 0.001 | 0.142 | 0.003 | 0.191 | 0.004 | 0.23 |
| 4-Hydroxypyridine | 0.011 | 1.553 | 0.005 | 1.738 | 0.086 | 1.293 | 0.118 | 1.477 | 0.355 | 1.297 | 0.153 | 1.267 | 0.391 | 0.899 | 0.465 | 1.25 |
| 5-Methyluridine | 0.040 | 0.586 | 0.563 | 1.046 | 0.199 | 2.567 | 0.143 | 0.741 | 0.908 | 1.524 | 0.223 | 1.823 | 0.668 | 0.889 | 0.088 | 0.42 |
| 9-Octadecenamide, (Z)- | 0.172 | 0.601 | 0.064 | 0.438 | 0.568 | 1.249 | 0.008 | 0.323 | 0.028 | 0.282 | 0.050 | 0.516 | 0.015 | 0.335 | 0.465 | 0.60 |
| Adenosine | 0.143 | 4.684 | 0.247 | 1.953 | 0.317 | 2.586 | 0.626 | 0.951 | 0.487 | 1.362 | 0.368 | 1.309 | 0.568 | 0.620 | 0.570 | 0.74 |
| Adenosine-5-monophosphate 1 | 0.001 | 7.333 | 0.001 | 16.036 | 0.003 | 26.774 | 0.008 | 11.150 | 0.005 | 13.068 | 0.002 | 15.333 | 0.086 | 2.286 | 0.019 | 3.94 |
| Alanine 1 | 0.283 | 1.064 | 0.021 | 1.562 | 0.153 | 1.472 | 0.283 | 1.098 | 0.021 | 1.634 | 0.017 | 1.490 | 0.475 | 1.136 | 0.465 | 0.91 |
| all-cis-4,7,10,13,16,19-Docosahexaenoic acid methyl ester | 0.118 | 1.793 | 0.037 | 2.250 | 0.568 | 0.809 | 0.118 | 1.763 | 0.132 | 0.694 | 0.711 | 0.714 | 0.086 | 0.501 | 0.570 | 0.65 |
| Alpha-Ketoglutaric acid | 0.626 | 1.367 | 0.203 | 1.663 | 0.063 | 1.945 | 0.845 | 1.334 | 0.021 | 4.244 | 0.017 | 2.535 | 0.153 | 1.654 | 0.570 | 1.26 |
| Alpha-Tocopherol | 0.143 | 1.642 | 0.728 | 1.179 | 0.886 | 1.120 | 0.696 | 1.305 | 0.203 | 0.779 | 0.560 | 1.251 | 0.475 | 1.187 | 0.372 | 0.64 |
| Arabitol | 0.097 | 0.554 | 0.487 | 0.880 | 0.153 | 0.550 | 0.032 | 0.589 | 0.005 | 0.399 | 0.005 | 0.396 | 0.003 | 0.276 | 0.004 | 0.30 |
| Arachidonic acid | 0.143 | 1.107 | 0.203 | 1.144 | 0.886 | 0.954 | 0.380 | 1.066 | 0.247 | 0.516 | 0.560 | 0.664 | 0.199 | 0.670 | 0.570 | 0.70 |
| Ascorbic acid | 0.329 | 1.904 | 0.247 | 1.622 | 0.317 | 0.423 | 0.495 | 1.519 | 0.132 | 0.608 | 0.634 | 1.331 | 0.063 | 0.218 | 0.042 | 0.34 |
| Asparagine 1 | 0.001 | 0.233 | 0.083 | 0.491 | 0.475 | 0.927 | 0.032 | 0.428 | 0.908 | 0.907 | 0.711 | 0.858 | 0.116 | 0.554 | 0.007 | 0.27 |
| Aspartic acid 1 | 0.770 | 1.477 | 0.247 | 1.904 | 0.886 | 1.155 | 0.380 | 1.488 | 0.298 | 1.753 | 0.017 | 2.676 | 0.032 | 3.508 | 0.123 | 0.66 |
| Benzoic acid | 0.064 | 1.416 | 0.132 | 1.510 | 0.032 | 1.700 | 0.097 | 1.248 | 0.083 | 1.509 | 0.223 | 1.316 | 0.317 | 1.368 | 0.062 | 1.68 |
| Beta-Alanine | 0.097 | 0.667 | 0.908 | 1.232 | 1.000 | 1.277 | 0.696 | 1.232 | 0.037 | 0.612 | 0.081 | 0.620 | 0.253 | 2.365 | 0.088 | 0.50 |
| Beta-Glycerolphosphate | 0.845 | 0.962 | 0.355 | 1.215 | 0.475 | 1.124 | 0.696 | 0.802 | 0.203 | 0.403 | 0.153 | 0.400 | 0.046 | 0.276 | 0.372 | 0.39 |
| Capric acid | 0.032 | 1.516 | 0.021 | 1.590 | 0.010 | 1.649 | 0.143 | 1.173 | 0.011 | 1.607 | 0.101 | 1.257 | 0.475 | 1.052 | 0.291 | 1.13 |
| Cholesterol | 0.097 | 0.761 | 0.298 | 0.984 | 0.063 | 1.084 | 0.558 | 0.958 | 0.203 | 0.805 | 0.560 | 0.825 | 0.022 | 0.647 | 0.372 | 0.83 |
| cis-5,8,11-Eicosatrienoic acid | 0.015 | 0.168 | 0.355 | 0.181 | 0.391 | 0.559 | 0.283 | 0.314 | 0.563 | 0.386 | 0.081 | 0.328 | 0.022 | 0.153 | 0.808 | 0.20 |
| cis-7,10,13,16-Docosatetraenoic acid | 0.380 | 0.673 | 0.908 | 0.902 | 0.199 | 0.692 | 0.558 | 0.763 | 0.728 | 0.982 | 0.368 | 1.090 | 0.475 | 0.751 | 0.570 | 0.78 |
| Cis-Aconitic acid | 0.011 | 0.532 | 0.105 | 0.610 | 0.116 | 0.638 | 0.032 | 0.529 | 0.028 | 0.564 | 0.023 | 0.554 | 0.032 | 0.466 | 0.291 | 0.72 |
| Citric acid | 0.001 | 0.122 | 0.004 | 0.272 | 0.010 | 0.205 | 0.002 | 0.176 | 0.008 | 0.319 | 0.007 | 0.346 | 0.010 | 0.367 | 0.012 | 0.20 |
| Citrulline 1 | 0.001 | 0.496 | 0.037 | 0.823 | 0.116 | 0.540 | 0.015 | 0.708 | 0.037 | 0.363 | 0.001 | 0.472 | 0.007 | 0.308 | 0.004 | 0.07 |
| Cysteine | 0.051 | 0.349 | 0.083 | 0.374 | 0.022 | 0.206 | 0.079 | 0.384 | 0.105 | 0.398 | 0.081 | 0.406 | 0.153 | 0.341 | 0.012 | 0.09 |
| D-Cellobiose | 0.011 | 0.348 | 0.203 | 0.706 | 0.668 | 0.760 | 0.283 | 0.672 | 0.298 | 0.681 | 0.081 | 0.433 | 0.046 | 0.530 | 0.123 | 0.32 |
| Dehydroascorbic acid 1 | 0.025 | 6.245 | 0.132 | 2.730 | 0.253 | 2.613 | 0.242 | 2.912 | 0.817 | 1.616 | 0.050 | 4.627 | 0.063 | 0.201 | 0.372 | 0.46 |
| D-Erythronolactone | 0.002 | 4.247 | 0.004 | 3.910 | 0.046 | 2.796 | 0.006 | 3.293 | 0.015 | 3.014 | 0.003 | 4.258 | 0.046 | 2.479 | 0.808 | 1.31 |
| D-Fructose 1 | 0.001 | 0.326 | 0.002 | 0.320 | 0.046 | 0.575 | 0.001 | 0.277 | 0.001 | 0.130 | 0.003 | 0.411 | 0.003 | 0.180 | 0.004 | 0.13 |
| D-Fructose-6-Phosphate | 0.001 | 0.104 | 0.001 | 0.120 | 0.003 | 0.166 | 0.002 | 0.144 | 0.002 | 0.132 | 0.010 | 0.134 | 0.003 | 0.128 | 0.012 | 0.15 |
| D-Gamma-Tocopherol | 0.011 | 2.998 | 0.028 | 2.222 | 0.046 | 4.085 | 0.040 | 3.044 | 0.083 | 3.305 | 0.005 | 2.957 | 0.004 | 4.050 | 0.372 | 1.66 |
| D-Glucose 1 | 0.001 | 0.014 | 0.001 | 0.013 | 0.046 | 0.069 | 0.002 | 0.010 | 0.004 | 0.014 | 0.001 | 0.032 | 0.046 | 0.147 | 0.028 | 0.03 |
| D-Glucose-6-Phosphate 1 | 0.001 | 0.058 | 0.001 | 0.110 | 0.003 | 0.140 | 0.002 | 0.220 | 0.002 | 0.154 | 0.013 | 0.165 | 0.007 | 0.208 | 0.019 | 0.20 |
| Dihydrocholesterol | 0.064 | 0.676 | 0.418 | 1.289 | 0.153 | 1.471 | 0.922 | 1.005 | 0.487 | 0.861 | 0.791 | 1.039 | 0.015 | 0.572 | 0.465 | 1.51 |
| D-Lyxose | 0.002 | 0.538 | 0.817 | 1.183 | 0.063 | 0.648 | 0.143 | 0.810 | 0.008 | 0.573 | 0.030 | 0.657 | 0.003 | 0.277 | 0.004 | 0.32 |
| D-Mannitol | 0.001 | 0.009 | 0.001 | 0.025 | 0.007 | 0.026 | 0.003 | 0.050 | 0.001 | 0.041 | 0.002 | 0.010 | 0.003 | 0.009 | 0.004 | 0.01 |
| D-myo-Inositol 4-Monophosphate | 0.003 | 1.984 | 0.005 | 2.083 | 0.004 | 2.785 | 0.011 | 1.899 | 0.028 | 1.572 | 0.039 | 1.789 | 0.317 | 1.571 | 0.167 | 2.30 |
| Docosahexaenoic acid | 0.626 | 0.808 | 0.908 | 0.898 | 0.253 | 0.641 | 0.283 | 0.697 | 0.049 | 0.506 | 0.223 | 0.764 | 0.116 | 0.679 | 0.223 | 0.59 |
| Docosanoic acid | 0.495 | 0.901 | 1.000 | 0.997 | 0.199 | 1.205 | 0.770 | 0.858 | 0.563 | 1.147 | 0.958 | 0.927 | 0.668 | 0.956 | 0.935 | 0.95 |
| D-Pinitol | 0.002 | 5.274 | 0.003 | 6.899 | 0.086 | 3.185 | 0.079 | 2.878 | 1.000 | 1.315 | 0.125 | 2.799 | 0.116 | 0.631 | 0.935 | 1.44 |
| D-Ribose-5-Phosphate | 0.040 | 0.380 | 0.021 | 0.339 | 0.253 | 0.495 | 0.015 | 0.281 | 0.028 | 0.244 | 0.017 | 0.303 | 0.032 | 0.311 | 0.042 | 0.44 |
| D-Ribulose5-phosphate | 0.002 | 0.048 | 0.011 | 0.086 | 0.116 | 0.160 | 0.015 | 0.223 | 0.049 | 0.133 | 0.007 | 0.083 | 0.022 | 0.224 | 0.062 | 0.36 |
| D-Sorbitol | 0.001 | 0.060 | 0.037 | 0.163 | 0.063 | 0.211 | 0.001 | 0.062 | 0.002 | 0.072 | 0.013 | 0.373 | 0.886 | 1.088 | 0.019 | 0.07 |
| Eicosanoic acid | 0.922 | 1.017 | 0.908 | 0.980 | 0.086 | 1.475 | 1.000 | 1.035 | 0.247 | 1.340 | 0.427 | 1.240 | 0.568 | 0.939 | 0.570 | 1.17 |
| Ethanolamine | 0.097 | 1.602 | 0.487 | 1.088 | 0.253 | 0.821 | 0.435 | 0.900 | 0.015 | 0.737 | 0.153 | 0.506 | 0.063 | 0.624 | 0.012 | 0.46 |
| Ethyl phosphoric acid | 0.032 | 9.215 | 0.015 | 11.559 | 0.046 | 37.731 | 0.118 | 2.473 | 0.049 | 5.488 | 0.491 | 1.863 | 0.475 | 0.934 | 0.935 | 1.34 |
| Ethylamine | 0.002 | 3.457 | 0.004 | 2.405 | 0.253 | 1.507 | 0.118 | 1.560 | 1.000 | 1.195 | 0.223 | 1.487 | 0.668 | 1.353 | 0.570 | 1.42 |
| Ethylene glycol | 0.006 | 0.681 | 0.247 | 0.852 | 0.153 | 0.830 | 0.005 | 0.695 | 0.028 | 0.778 | 0.017 | 0.782 | 0.032 | 0.635 | 0.808 | 1.13 |
| Fumaric acid | 0.626 | 0.967 | 0.643 | 1.038 | 0.775 | 1.047 | 1.000 | 1.071 | 0.817 | 1.081 | 0.427 | 1.041 | 0.668 | 1.104 | 0.223 | 0.43 |
| Galactitol | 0.770 | 0.926 | 0.064 | 1.839 | 0.253 | 1.311 | 0.770 | 1.071 | 0.064 | 2.078 | 0.013 | 2.715 | 0.153 | 2.499 | 0.167 | 0.56 |
| Glutamic acid 1 | 0.008 | 0.404 | 0.247 | 0.844 | 0.116 | 0.597 | 0.011 | 0.532 | 0.028 | 1.450 | 0.101 | 1.740 | 0.391 | 1.387 | 0.012 | 0.27 |
| Glyceric acid | 0.064 | 0.345 | 0.203 | 0.514 | 0.668 | 0.700 | 0.329 | 0.643 | 1.000 | 0.824 | 0.560 | 0.661 | 0.391 | 1.247 | 0.685 | 0.87 |
| Glycerol | 0.003 | 0.434 | 0.005 | 0.614 | 0.007 | 0.579 | 0.001 | 0.504 | 0.028 | 0.498 | 0.005 | 0.446 | 0.022 | 0.308 | 0.004 | 0.33 |
| Glycerol 1-palmitate | 0.558 | 0.907 | 0.247 | 1.102 | 0.153 | 1.341 | 0.696 | 0.867 | 0.165 | 0.587 | 0.186 | 0.630 | 0.063 | 0.647 | 0.465 | 0.79 |
| Glycerol 3-phosphate | 0.329 | 0.653 | 0.563 | 0.788 | 1.000 | 1.025 | 0.143 | 0.531 | 0.049 | 0.120 | 0.050 | 0.135 | 0.032 | 0.053 | 0.123 | 0.28 |
| Glycine 1 | 0.696 | 1.028 | 0.083 | 1.811 | 0.391 | 0.665 | 0.097 | 1.651 | 0.817 | 1.095 | 0.634 | 1.052 | 0.475 | 1.126 | 0.088 | 0.62 |
| Glycolic acid | 0.079 | 1.429 | 0.015 | 1.824 | 0.007 | 2.284 | 0.003 | 2.124 | 0.002 | 2.454 | 0.017 | 1.813 | 0.022 | 1.971 | 0.372 | 1.17 |
| Heptadecanoic acid | 0.118 | 0.768 | 0.355 | 0.842 | 0.317 | 1.165 | 0.205 | 0.715 | 0.028 | 1.777 | 0.002 | 1.809 | 0.199 | 1.464 | 0.808 | 0.82 |
| Heptanoic acid | 0.006 | 1.832 | 0.011 | 2.227 | 0.007 | 2.195 | 0.097 | 1.660 | 0.049 | 1.268 | 0.711 | 1.048 | 0.668 | 1.183 | 0.012 | 2.10 |
| Hexadecanoic acid, methyl ester | 0.015 | 4.020 | 0.064 | 3.387 | 0.253 | 2.403 | 0.097 | 2.496 | 0.817 | 1.451 | 0.064 | 3.068 | 0.391 | 1.556 | 0.935 | 1.19 |
| Hexanoic acid | 0.242 | 1.365 | 0.015 | 1.841 | 0.003 | 2.265 | 0.040 | 1.518 | 0.064 | 1.348 | 0.711 | 1.161 | 0.391 | 1.419 | 0.088 | 2.26 |
| Hexyl alcohol | 0.143 | 1.319 | 0.064 | 1.542 | 0.032 | 1.590 | 0.079 | 1.312 | 0.083 | 1.435 | 0.153 | 1.284 | 0.391 | 1.264 | 0.042 | 1.80 |
| Hydroxylamine | 0.696 | 0.604 | 0.298 | 5.247 | 0.153 | 9.104 | 0.380 | 3.778 | 0.049 | 8.374 | 0.634 | 3.612 | 0.032 | 8.341 | 0.012 | 12.90 |
| Hypotaurine | 0.005 | 0.206 | 0.247 | 2.088 | 0.086 | 0.242 | 0.770 | 0.912 | 0.003 | 0.145 | 0.010 | 0.251 | 0.046 | 0.373 | 0.028 | 0.19 |
| Inosine | 0.097 | 1.112 | 0.643 | 0.999 | 0.063 | 0.667 | 0.495 | 0.612 | 0.165 | 0.644 | 0.491 | 0.819 | 0.116 | 0.419 | 0.123 | 0.40 |
| Isocitrate | 0.001 | 0.111 | 0.002 | 0.227 | 0.003 | 0.190 | 0.002 | 0.224 | 0.003 | 0.280 | 0.005 | 0.204 | 0.007 | 0.333 | 0.004 | 0.25 |
| Isoleucine 1 | 0.001 | 0.569 | 0.004 | 0.614 | 0.015 | 0.746 | 0.005 | 0.673 | 0.008 | 0.547 | 0.007 | 0.747 | 0.086 | 0.738 | 0.004 | 0.39 |
| Lactic acid | 0.008 | 0.608 | 0.418 | 0.931 | 0.116 | 0.746 | 0.064 | 0.775 | 0.064 | 0.590 | 0.223 | 0.748 | 0.022 | 0.558 | 0.004 | 0.20 |
| Lactobionic acid | 0.001 | 0.012 | 0.001 | 0.025 | 0.003 | 0.046 | 0.001 | 0.021 | 0.001 | 0.035 | 0.001 | 0.037 | 0.003 | 0.019 | 0.004 | 0.02 |
| Lactose | 0.922 | 1.278 | 0.728 | 1.018 | 0.568 | 0.741 | 0.696 | 0.914 | 0.064 | 0.512 | 0.560 | 0.724 | 0.475 | 0.721 | 0.062 | 0.34 |
| Lauric acid | 0.922 | 0.924 | 0.563 | 1.017 | 0.391 | 1.166 | 0.626 | 0.930 | 0.908 | 0.975 | 0.791 | 0.849 | 0.775 | 1.059 | 0.223 | 1.28 |
| Leucine 1 | 0.001 | 0.514 | 0.002 | 0.593 | 0.032 | 0.816 | 0.001 | 0.638 | 0.008 | 0.454 | 0.001 | 0.581 | 0.015 | 0.584 | 0.004 | 0.42 |
| L-Fucose | 0.011 | 0.476 | 0.037 | 0.521 | 0.153 | 0.586 | 0.019 | 0.481 | 0.011 | 0.385 | 0.039 | 0.402 | 0.153 | 0.618 | 0.012 | 0.35 |
| Linoleic acid | 0.380 | 0.857 | 0.105 | 0.520 | 0.153 | 0.591 | 0.064 | 0.484 | 0.165 | 0.579 | 0.634 | 0.888 | 0.668 | 0.739 | 0.291 | 0.65 |
| L-Threonic acid | 0.001 | 3.843 | 0.002 | 6.315 | 0.003 | 6.988 | 0.001 | 12.324 | 0.001 | 16.258 | 0.003 | 4.719 | 0.003 | 15.784 | 0.012 | 3.72 |
| Lysine 1 | 0.001 | 0.392 | 0.002 | 0.651 | 0.668 | 1.012 | 0.003 | 0.749 | 0.028 | 0.683 | 0.017 | 0.640 | 0.046 | 0.580 | 0.062 | 0.37 |
| Malic acid | 0.205 | 0.526 | 0.908 | 0.794 | 0.153 | 0.677 | 0.143 | 0.675 | 0.643 | 0.843 | 0.874 | 0.864 | 0.668 | 1.087 | 0.088 | 0.39 |
| Maltose | 0.008 | 0.359 | 0.165 | 0.742 | 0.668 | 0.802 | 0.283 | 0.706 | 0.355 | 0.757 | 0.081 | 0.421 | 0.032 | 0.595 | 0.123 | 0.34 |
| Maltotriose | 0.006 | 0.268 | 0.165 | 0.524 | 1.000 | 0.904 | 0.205 | 0.570 | 0.298 | 0.657 | 0.064 | 0.430 | 0.063 | 0.395 | 0.062 | 0.46 |
| Mannose 1 | 0.001 | 0.025 | 0.001 | 0.051 | 0.003 | 0.110 | 0.001 | 0.046 | 0.001 | 0.051 | 0.001 | 0.041 | 0.003 | 0.196 | 0.004 | 0.08 |
| Meso-Erythritol | 0.001 | 0.427 | 0.001 | 0.334 | 0.003 | 0.411 | 0.001 | 0.252 | 0.001 | 0.288 | 0.001 | 0.323 | 0.003 | 0.414 | 0.004 | 0.18 |
| Methionine 1 | 0.005 | 0.549 | 0.165 | 0.718 | 0.886 | 1.017 | 0.172 | 0.695 | 0.037 | 0.563 | 0.010 | 0.541 | 0.253 | 0.840 | 0.012 | 0.45 |
| Methyl stearate | 0.770 | 1.123 | 0.247 | 0.887 | 0.568 | 0.966 | 0.242 | 0.756 | 0.908 | 1.153 | 0.560 | 1.172 | 0.199 | 0.731 | 0.088 | 0.50 |
| Methyl-9-Octadecenoate | 0.380 | 0.917 | 0.165 | 0.793 | 0.253 | 0.810 | 0.205 | 0.834 | 0.064 | 0.663 | 0.427 | 0.794 | 0.116 | 0.514 | 0.291 | 0.67 |
| Mimosine | 0.006 | 0.484 | 0.037 | 0.769 | 0.775 | 1.112 | 0.015 | 0.666 | 0.011 | 0.563 | 0.023 | 0.533 | 0.015 | 0.456 | 0.012 | 0.43 |
| mono-Methyl phosphate | 0.435 | 1.046 | 0.563 | 0.989 | 0.775 | 0.945 | 0.922 | 0.900 | 0.728 | 0.918 | 0.368 | 1.145 | 0.116 | 0.692 | 0.223 | 0.67 |
| Monostearin | 0.329 | 1.126 | 0.817 | 0.956 | 0.046 | 1.329 | 0.696 | 1.014 | 0.643 | 1.067 | 0.186 | 1.153 | 0.775 | 1.011 | 0.935 | 1.03 |
| Myo-Inositol | 0.003 | 2.695 | 0.011 | 3.444 | 0.032 | 3.113 | 0.040 | 1.833 | 0.011 | 0.452 | 0.125 | 0.521 | 0.007 | 0.197 | 0.042 | 0.62 |
| Myristic acid | 0.380 | 0.816 | 0.487 | 1.206 | 0.775 | 0.942 | 1.000 | 0.983 | 0.643 | 0.777 | 0.186 | 0.670 | 0.475 | 0.777 | 0.465 | 1.19 |
| N-Acetyl-DL-serine | 0.380 | 0.978 | 0.083 | 3.204 | 0.568 | 1.285 | 0.380 | 1.531 | 0.011 | 2.589 | 0.010 | 2.519 | 0.668 | 1.388 | 0.808 | 1.70 |
| N-Acetyl-D-Mannosamine 1 | 0.626 | 0.838 | 0.817 | 0.903 | 0.116 | 0.627 | 0.922 | 0.864 | 0.247 | 0.735 | 0.050 | 0.495 | 0.086 | 1.780 | 0.935 | 0.92 |
| N-Acetyl-L-Aspartic acid 1 | 0.696 | 1.033 | 0.563 | 1.293 | 0.775 | 1.346 | 0.626 | 0.697 | 0.563 | 0.892 | 0.874 | 1.007 | 0.063 | 0.302 | 0.088 | 0.32 |
| N-Acetyl-L-Glutamic acid | 0.242 | 1.190 | 0.005 | 2.112 | 0.391 | 1.137 | 0.626 | 1.008 | 0.037 | 1.741 | 0.101 | 1.436 | 0.022 | 0.371 | 0.223 | 0.54 |
| N-Acetylneuraminic acid | 0.696 | 0.929 | 0.247 | 1.259 | 0.775 | 0.723 | 0.329 | 1.185 | 0.083 | 1.601 | 0.874 | 0.782 | 0.003 | 0.343 | 0.028 | 0.30 |
| N-Carboxyglycine | 0.283 | 1.408 | 0.165 | 1.619 | 0.063 | 1.595 | 0.242 | 1.355 | 0.165 | 1.462 | 0.427 | 1.216 | 0.153 | 1.399 | 0.372 | 1.31 |
| Nicotinamide | 0.143 | 1.482 | 0.132 | 1.550 | 0.668 | 1.195 | 0.696 | 1.265 | 1.000 | 0.997 | 0.634 | 1.158 | 0.668 | 1.128 | 0.088 | 0.62 |
| Nonanoic acid | 0.118 | 1.523 | 0.203 | 1.384 | 0.015 | 1.718 | 0.283 | 1.188 | 0.064 | 1.324 | 0.491 | 1.210 | 0.475 | 1.216 | 0.223 | 1.37 |
| n-Pentadecanoic acid | 0.626 | 1.411 | 0.298 | 1.237 | 0.668 | 1.024 | 1.000 | 0.923 | 0.487 | 0.779 | 0.491 | 1.245 | 0.568 | 1.101 | 0.223 | 0.76 |
| Octanoic acid | 0.011 | 1.853 | 0.008 | 1.861 | 0.003 | 2.049 | 0.025 | 1.637 | 0.004 | 3.008 | 0.004 | 1.868 | 0.022 | 1.579 | 0.028 | 1.47 |
| Oleic acid | 0.845 | 0.999 | 0.418 | 0.844 | 0.253 | 0.797 | 0.283 | 0.800 | 0.908 | 0.979 | 0.491 | 1.084 | 0.253 | 0.592 | 0.123 | 0.73 |
| O-Phosphocolamine | 0.002 | 5.080 | 0.021 | 2.747 | 0.199 | 1.749 | 0.064 | 2.018 | 0.165 | 1.667 | 0.030 | 2.133 | 0.253 | 1.399 | 0.935 | 1.15 |
| Ornithine 2 | 0.008 | 0.459 | 0.247 | 0.734 | 0.391 | 1.478 | 0.242 | 0.651 | 0.049 | 1.836 | 0.368 | 1.708 | 0.253 | 1.365 | 0.808 | 1.24 |
| Oxalic acid | 0.001 | 6.588 | 0.001 | 9.228 | 0.003 | 4.929 | 0.001 | 8.942 | 0.001 | 8.630 | 0.001 | 5.361 | 0.003 | 6.229 | 0.004 | 5.38 |
| Palmitelaidic acid | 0.558 | 1.421 | 0.165 | 2.116 | 0.568 | 1.439 | 0.922 | 1.350 | 0.001 | 0.496 | 0.003 | 0.464 | 0.391 | 0.904 | 0.685 | 0.96 |
| Palmitic acid | 0.283 | 1.155 | 0.165 | 1.373 | 0.116 | 1.425 | 0.380 | 1.089 | 0.908 | 0.987 | 0.791 | 0.853 | 1.000 | 0.932 | 0.167 | 1.21 |
| Palmitoleic acid | 0.558 | 1.101 | 0.064 | 1.680 | 0.886 | 1.092 | 0.283 | 1.487 | 0.037 | 0.631 | 0.003 | 0.653 | 0.063 | 0.791 | 0.372 | 0.67 |
| Pantothenic acid | 0.015 | 0.733 | 0.728 | 1.299 | 0.199 | 0.613 | 0.001 | 0.658 | 0.728 | 0.923 | 0.125 | 1.723 | 0.199 | 0.739 | 0.004 | 0.27 |
| Phenylalanine 1 | 0.001 | 0.414 | 0.001 | 0.534 | 0.668 | 0.958 | 0.005 | 0.515 | 0.015 | 0.428 | 0.001 | 0.525 | 0.046 | 0.673 | 0.062 | 0.47 |
| Phosphate | 0.040 | 1.910 | 0.028 | 1.853 | 0.199 | 1.674 | 0.205 | 1.593 | 0.487 | 1.401 | 0.153 | 1.623 | 0.886 | 1.149 | 0.465 | 0.91 |
| Phosphoenolpyruvic acid | 0.380 | 0.194 | 0.298 | 0.208 | 0.317 | 0.311 | 0.845 | 0.599 | 0.643 | 0.680 | 0.427 | 0.223 | 0.391 | 2.274 | 0.808 | 0.70 |
| Porphine | 0.006 | 1.739 | 0.005 | 1.970 | 0.010 | 2.001 | 0.003 | 1.800 | 0.028 | 1.662 | 0.030 | 1.629 | 0.391 | 1.290 | 0.012 | 2.02 |
| Proline 1 | 0.025 | 0.768 | 0.049 | 0.657 | 0.199 | 0.828 | 0.064 | 0.699 | 0.728 | 1.140 | 0.266 | 1.385 | 0.886 | 1.034 | 0.004 | 0.31 |
| Pyroglutamic acid 2 | 0.001 | 0.317 | 0.132 | 0.749 | 0.022 | 0.616 | 0.002 | 0.693 | 0.908 | 1.012 | 0.368 | 1.228 | 0.568 | 0.929 | 0.004 | 0.25 |
| Pyrophosphate | 0.015 | 5.515 | 0.049 | 5.110 | 0.086 | 3.068 | 0.011 | 3.915 | 0.037 | 3.461 | 0.039 | 4.547 | 0.116 | 3.568 | 0.223 | 1.09 |
| Pyrrole-2-carboxylic acid | 0.002 | 1.769 | 0.003 | 2.029 | 0.046 | 1.463 | 0.051 | 1.617 | 0.203 | 1.286 | 0.064 | 1.407 | 0.886 | 0.921 | 0.088 | 1.41 |
| Pyruvic acid | 0.040 | 1.402 | 0.037 | 2.731 | 0.010 | 6.264 | 0.040 | 2.909 | 0.004 | 6.451 | 0.007 | 5.956 | 0.116 | 2.704 | 0.935 | 0.99 |
| Raffinose | 0.001 | 0.070 | 0.002 | 0.113 | 0.010 | 0.201 | 0.002 | 0.096 | 0.003 | 0.158 | 0.001 | 0.079 | 0.007 | 0.123 | 0.007 | 0.16 |
| Ribitol | 0.097 | 0.677 | 0.165 | 0.840 | 0.032 | 0.449 | 0.025 | 0.564 | 0.003 | 0.388 | 0.039 | 0.464 | 0.086 | 0.511 | 0.007 | 0.21 |
| Ribose | 0.001 | 0.150 | 0.001 | 0.182 | 0.010 | 0.281 | 0.001 | 0.236 | 0.004 | 0.136 | 0.001 | 0.115 | 0.010 | 0.240 | 0.007 | 0.28 |
| Serine 2 | 0.001 | 0.551 | 0.064 | 0.846 | 0.317 | 0.852 | 0.143 | 0.812 | 0.203 | 0.792 | 0.153 | 0.683 | 0.046 | 0.741 | 0.007 | 0.59 |
| Stearic acid | 0.495 | 0.932 | 0.817 | 0.969 | 0.391 | 1.124 | 0.380 | 0.835 | 0.728 | 1.016 | 0.368 | 1.046 | 0.568 | 0.841 | 0.808 | 0.74 |
| Succinic acid | 0.118 | 0.374 | 0.643 | 1.020 | 0.568 | 1.345 | 0.380 | 0.568 | 0.487 | 0.649 | 0.874 | 0.692 | 0.116 | 0.576 | 0.685 | 0.81 |
| Sucrose | 0.172 | 1.962 | 0.037 | 3.036 | 0.022 | 5.768 | 0.097 | 2.817 | 0.355 | 1.795 | 0.958 | 1.435 | 0.568 | 0.882 | 0.123 | 4.80 |
| Threonine 1 | 0.006 | 0.657 | 0.064 | 0.813 | 0.116 | 0.816 | 0.005 | 0.629 | 0.487 | 1.155 | 0.560 | 1.420 | 0.317 | 0.943 | 0.004 | 0.36 |
| Trans-4-Hydroxyproline 1 | 1.000 | 1.085 | 0.203 | 0.608 | 0.775 | 1.269 | 0.558 | 0.811 | 0.037 | 2.404 | 0.186 | 2.170 | 0.568 | 0.969 | 0.465 | 0.64 |
| trans-9-Octadecenoic acid | 0.064 | 1.195 | 0.028 | 1.459 | 0.886 | 0.857 | 0.242 | 1.314 | 0.563 | 0.816 | 0.560 | 0.878 | 0.475 | 0.809 | 0.372 | 1.26 |
| Tryptophan 1 | 0.006 | 0.602 | 0.247 | 0.748 | 0.775 | 0.908 | 0.079 | 0.666 | 0.028 | 0.464 | 0.023 | 0.587 | 0.116 | 0.603 | 0.007 | 0.35 |
| Tyrosine 1 | 0.001 | 0.580 | 0.021 | 0.772 | 0.775 | 1.071 | 0.064 | 0.797 | 0.008 | 0.552 | 0.003 | 0.665 | 0.032 | 0.713 | 0.042 | 0.59 |
| Uracil | 0.495 | 0.979 | 0.298 | 0.775 | 0.015 | 0.492 | 0.205 | 0.623 | 0.004 | 0.265 | 0.023 | 0.303 | 0.116 | 0.558 | 0.007 | 0.27 |
| Urea | 0.051 | 1.842 | 0.021 | 1.841 | 0.199 | 1.688 | 0.205 | 1.616 | 0.355 | 1.397 | 0.125 | 1.618 | 0.668 | 1.273 | 0.465 | 0.91 |
| Uric acid 2 | 0.001 | 5.497 | 0.021 | 4.800 | 0.007 | 12.337 | 0.097 | 2.334 | 0.203 | 2.182 | 0.368 | 2.021 | 0.317 | 1.826 | 0.019 | 6.78 |
| Uridine 2 | 0.015 | 0.363 | 0.132 | 0.503 | 0.475 | 0.772 | 0.064 | 0.451 | 0.247 | 0.623 | 0.560 | 0.629 | 0.568 | 1.544 | 0.062 | 0.46 |
| Valine 1 | 0.019 | 0.946 | 0.165 | 0.970 | 0.253 | 0.983 | 0.143 | 0.967 | 0.037 | 0.760 | 0.186 | 0.978 | 0.475 | 0.944 | 0.004 | 0.59 |
| Xylitol | 0.001 | 0.336 | 0.021 | 0.440 | 0.086 | 0.624 | 0.001 | 0.338 | 0.005 | 0.240 | 0.005 | 0.455 | 1.000 | 0.997 | 0.007 | 0.32 |
| Zymosterol | 0.435 | 3.124 | 0.005 | 9.625 | 0.199 | 3.373 | 0.064 | 4.021 | 0.037 | 4.901 | 0.023 | 6.980 | 0.086 | 4.755 | 0.088 | 6.56 |
