# Supplementary material for: Metabolic profiling reveals distinct metabolic alterations in different subtypes of pituitary adenomas and confers therapeutic targets
Source: J Transl Med. 2019 Aug 28;17:291. doi: 10.1186/s12967-019-2042-9 (PMC6712670; doi:10.1186/s12967-019-2042-9)
Supplement: Supplementary file 6 — Additional file 6: Table S3. A summary of the protein-metabolite joint pathways from enrichment analysis and topology analysis. [file 12967_2019_2042_MOESM6_ESM.docx]

| Additional Table S3 a summary of the protein-metabolite joint pathways from enrichment analysis and topology analysis | | | | | | | |
| --- | --- | --- | --- | --- | --- | --- | --- |
| Pathway | Total | Expected | Hits | P.Value | Topology | PVal.Z | Topo.Z |
| Aminoacyl-tRNA biosynthesis | 87 | 8.0916 | 20 | 6.25E-05 | 0.47826 | 4.3095 | 0.39591 |
| Propanoate metabolism | 52 | 4.8363 | 14 | 0.000147 | 1.0244 | 3.8788 | 1.945 |
| Pentose phosphate pathway | 48 | 4.4643 | 12 | 0.000946 | 1.0976 | 2.945 | 2.1526 |
| Glycolysis / Gluconeogenesis | 91 | 8.4636 | 17 | 0.002963 | 0.94915 | 2.372 | 1.7316 |
| Valine, leucine and isoleucine biosynthesis | 13 | 1.2091 | 5 | 0.004497 | 1.1818 | 2.1625 | 2.3916 |
| beta-Alanine metabolism | 50 | 4.6503 | 11 | 0.00465 | 0.83333 | 2.1458 | 1.4031 |
| Valine, leucine and isoleucine degradation | 82 | 7.6265 | 15 | 0.006476 | 0.4 | 1.9795 | 0.17392 |
| Citrate cycle (TCA cycle) | 50 | 4.6503 | 10 | 0.013805 | 1.2045 | 1.5995 | 2.456 |
| Galactose metabolism | 55 | 5.1154 | 9 | 0.062177 | 0.5102 | 0.84404 | 0.48652 |
| Arginine and proline metabolism | 102 | 9.4867 | 14 | 0.082406 | 0.52688 | 0.70263 | 0.53382 |
| Glyoxylate and dicarboxylate metabolism | 53 | 4.9293 | 8 | 0.11146 | 0.54 | 0.55105 | 0.57103 |
| Fructose and mannose metabolism | 55 | 5.1154 | 8 | 0.13107 | 0.56757 | 0.46967 | 0.64923 |
| Lysine biosynthesis | 7 | 0.65105 | 2 | 0.13225 | 1.125 | 0.46518 | 2.2304 |
| Starch and sucrose metabolism | 78 | 7.2545 | 10 | 0.1808 | 0.72222 | 0.30819 | 1.0879 |
| D-Glutamine and D-glutamate metabolism | 9 | 0.83706 | 2 | 0.20097 | 0.71429 | 0.2551 | 1.0654 |
| Phenylalanine, tyrosine and tryptophan biosynthesis | 9 | 0.83706 | 2 | 0.20097 | 1.2 | 0.2551 | 2.4431 |
| Histidine metabolism | 44 | 4.0923 | 6 | 0.21939 | 0.1875 | 0.21107 | -0.42885 |
| Pantothenate and CoA biosynthesis | 35 | 3.2552 | 5 | 0.2204 | 0.41935 | 0.20877 | 0.22882 |
| Pyruvate metabolism | 64 | 5.9524 | 8 | 0.23838 | 0.52174 | 0.1694 | 0.51923 |
| Phenylalanine metabolism | 29 | 2.6972 | 4 | 0.28078 | 0.68182 | 0.08722 | 0.9733 |
| Glycerolipid metabolism | 72 | 6.6965 | 8 | 0.3521 | 0.45946 | -0.0264 | 0.34258 |
| Pentose and glucuronate interconversions | 52 | 4.8363 | 6 | 0.35285 | 0.44828 | -0.02746 | 0.31085 |
| Lysine degradation | 73 | 6.7895 | 8 | 0.36694 | 0.37209 | -0.04713 | 0.094759 |
| Ubiquinone and other terpenoid-quinone biosynthesis | 5 | 0.46503 | 1 | 0.38666 | 0.25 | -0.07341 | -0.25156 |
| Glutathione metabolism | 75 | 6.9755 | 8 | 0.39678 | 0.50943 | -0.08638 | 0.48433 |
| Ascorbate and aldarate metabolism | 35 | 3.2552 | 4 | 0.4129 | 0.33333 | -0.10636 | -0.01518 |
| Alanine, aspartate and glutamate metabolism | 56 | 5.2084 | 6 | 0.42266 | 0.4898 | -0.11809 | 0.42863 |
| Butanoate metabolism | 47 | 4.3713 | 5 | 0.4477 | 0.34375 | -0.14698 | 0.014363 |
| One carbon pool by folate | 28 | 2.6042 | 3 | 0.49139 | 0.53333 | -0.19373 | 0.55212 |
| Biotin metabolism | 7 | 0.65105 | 1 | 0.49586 | 0.076923 | -0.19828 | -0.7425 |
| Butirosin and neomycin biosynthesis | 7 | 0.65105 | 1 | 0.49586 | 0.66667 | -0.19828 | 0.93033 |
| Taurine and hypotaurine metabolism | 18 | 1.6741 | 2 | 0.51026 | 0.33333 | -0.21265 | -0.01518 |
| Fatty acid metabolism | 83 | 7.7195 | 8 | 0.51525 | 1.165 | -0.21753 | 2.344 |
| Amino sugar and nucleotide sugar metabolism | 84 | 7.8125 | 8 | 0.52964 | 0.51852 | -0.23136 | 0.5101 |
| Tryptophan metabolism | 80 | 7.4405 | 7 | 0.62928 | 0.2375 | -0.31789 | -0.28702 |
| Cyanoamino acid metabolism | 12 | 1.1161 | 1 | 0.6916 | 0.5 | -0.3653 | 0.45757 |
| Cysteine and methionine metabolism | 63 | 5.8594 | 5 | 0.71438 | 0.25455 | -0.38157 | -0.23867 |
| Nicotinate and nicotinamide metabolism | 39 | 3.6273 | 3 | 0.71986 | 0.37838 | -0.38541 | 0.11259 |
| Vitamin B6 metabolism | 15 | 1.3951 | 1 | 0.77056 | 0.3 | -0.41957 | -0.10974 |
| Riboflavin metabolism | 20 | 1.8601 | 1 | 0.86007 | 0.15385 | -0.47474 | -0.52431 |
| Terpenoid backbone biosynthesis | 39 | 3.6273 | 2 | 0.89238 | 0.11429 | -0.49326 | -0.63652 |
| Glycine, serine and threonine metabolism | 68 | 6.3244 | 4 | 0.89341 | 0.15873 | -0.49383 | -0.51045 |
| Porphyrin and chlorophyll metabolism | 70 | 6.5105 | 4 | 0.9061 | 0.14894 | -0.50091 | -0.53823 |
| Glycosphingolipid biosynthesis - lacto and neolacto series | 26 | 2.4182 | 1 | 0.92289 | 0.033898 | -0.51013 | -0.86454 |
| Biosynthesis of unsaturated fatty acids | 27 | 2.5112 | 1 | 0.93019 | 0.03125 | -0.51409 | -0.87205 |
| Selenocompound metabolism | 33 | 3.0692 | 1 | 0.96166 | 0.14286 | -0.53079 | -0.55548 |
| alpha-Linolenic acid metabolism | 34 | 3.1622 | 1 | 0.96531 | 0.3 | -0.53269 | -0.10974 |
| Steroid biosynthesis | 54 | 5.0224 | 2 | 0.96876 | 0.10294 | -0.53448 | -0.6687 |
| Fatty acid elongation | 57 | 5.3014 | 2 | 0.97585 | 1 | -0.53814 | 1.8758 |
| Arachidonic acid metabolism | 100 | 9.3006 | 4 | 0.98863 | 0.23077 | -0.54468 | -0.30611 |
| N-Glycan biosynthesis | 50 | 4.6503 | 1 | 0.99308 | 0.028571 | -0.54693 | -0.87965 |
| Inositol phosphate metabolism | 90 | 8.3706 | 3 | 0.99335 | 0.09375 | -0.54707 | -0.69477 |
| Ether lipid metabolism | 51 | 4.7433 | 1 | 0.99375 | 0.068966 | -0.54727 | -0.76507 |
| Tyrosine metabolism | 80 | 7.4405 | 2 | 0.9969 | 0.27586 | -0.54885 | -0.1782 |
| Primary bile acid biosynthesis | 63 | 5.8594 | 1 | 0.99816 | 0.085106 | -0.54949 | -0.71929 |
| Pyrimidine metabolism | 142 | 13.207 | 5 | 0.99846 | 0.31858 | -0.54964 | -0.05702 |
| Glycerophospholipid metabolism | 119 | 11.068 | 3 | 0.99946 | 0.16 | -0.55014 | -0.50685 |
| Drug metabolism - other enzymes | 77 | 7.1615 | 1 | 0.99957 | 0.035088 | -0.5502 | -0.86117 |
| Purine metabolism | 234 | 21.764 | 9 | 0.99984 | 0.3908 | -0.55033 | 0.14783 |
| Metabolism of xenobiotics by cytochrome P450 | 139 | 12.928 | 2 | 0.99999 | 0.10909 | -0.55041 | -0.65126 |
| Drug metabolism - cytochrome P450 | 127 | 11.812 | 1 | 1 | 0.055556 | -0.55041 | -0.80311 |
