# Supplementary material for: Metabolic profiling reveals distinct metabolic alterations in different subtypes of pituitary adenomas and confers therapeutic targets
Source: J Transl Med. 2019 Aug 28;17:291. doi: 10.1186/s12967-019-2042-9 (PMC6712670; doi:10.1186/s12967-019-2042-9)
Supplement: Supplementary file 7 — Additional file 7: Table S4. The H-Score of IDH2 in different subtypes of pituitary adenomas. [file 12967_2019_2042_MOESM7_ESM.docx]

Additional Table S4 The H-Score of IDH2 in different subtypes of pituitary adenomas

| **Subtypes of pituitary adenomas** | **No.** | **H-Score** |
| --- | --- | --- |
| Corticotroph adenomas | 10 | 10.47±13.2 |
| Gonadotroph adenomas | 10 | 50.17±21.3 |
| Lactotroph adenomas | 5 | 41.72±17.76 |
| Mammosomatotroph adenomas | 8 | 60.1±23.41 |
| **Somatotroph adenomas** | **8** | **79.5±26.45** |
| Null cell adenomas | 5 | 45.63±19.26 |
| Oncocytoma | 10 | 40.63±17.72 |

Note: we measured the protein expression of IDH2 using the immunostaining. The H-score was obtained by multiplying the staining intensity with a constant to adjust the mean to the strongest staining [H-score = 3× (percentage of strong staining); 1.0 (% weak), 2.0 (% moderate), 3.0 (% strong)] to give a score ranging from 0–300.
